# Echocardiogram Testing in Patients with Post-COVID-19 Condition: A Systematic Review and Meta-Analysis

**DOI:** 10.3390/jcm15124643

**Published:** 2026-06-15

**Authors:** Jana Khawandi, Ali Choaib, Muayad Azzam, Gavin Y. Oudit, Khushi Patel, Jamil Nazzal, Aseel Al Khader, Hassan Kawtharany, Mohammad Amin Al Zabibi, Jood Ahmad, Husam Kivan, Sahar Al Hussein, Aqeeb Ur Rehman, Alain Piché, Andrea Vasquez Camargo, Candace McNaughton, Grace Y. Lam, Rejina Kamrul, Samia Afzal, Holger J. Schunemann, Wojtek Wiercioch, Robby Nieuwlaat, Romina Brignardello-Petersen, Emilia Liana Falcone, Reem A. Mustafa

**Affiliations:** 1 Evidence-Based Practice and Impact Center, Department of Internal Medicine, University of Kansas Medical Center, Kansas City, KS 66103, USA; 2 Division of Cardiology, Department of Medicine, Faculty of Medicine & Dentistry, University of Alberta, Edmonton, AB T6G 2R3, Canada; 3 Mazankowski Alberta Heart Institute, Edmonton, AB T6G 2R3, Canada; 4 University of Missouri-Kansas City School of Medicine, Kansas City, MO 64108, USA; 5 Faculty of Medicine, University of Jordan, Amman 11942, Jordan; 6 Division of Nephrology, Department of Medicine, Washington University in St. Louis, St. Louis, MO 63110, USA; 7 Department of Internal Medicine, University of Alabama at Birmingham, Birmingham, AL 35294, USA; 8 Département de Microbiologie et Maladies Infectieuses, Faculté de Médecine et des Sciences de la Santé, Université de Sherbrooke, Sherbrooke, QC J1E 4K8, Canada; 9 Department of Family Medicine, University of Saskatchewan, Regina, SK S4P 2S5, Canada; 10 Department of Medicine, University of Toronto, Sunnybrook Research Institute, Toronto, ON M4N 3M5, Canada; 11 Sunnybrook Research Institute, Toronto, ON M4N 3M5, Canada; 12 Division of Pulmonary Medicine, Department of Medicine, University of Alberta, Edmonton, AB T6G 2R7, Canada; 13 Alberta Respiratory Centre, University of Alberta, Edmonton, AB T6G 2R7, Canada; 14 Women and Children’s Health Research Institute, University of Alberta, Edmonton, AB T6G 2B7, Canada; 15 Health Information and Research Unit, McMaster University, Hamilton, ON L8S 4L8, Canada; 16 Department of Health Research Methods, Evidence, and Impact, McMaster University, Hamilton, ON L8S 4L8, Canada; 17 World Health Organization Collaborating Center for Infectious Diseases, Research Methods and Recommendations, McMaster University, Hamilton, ON L8S 4L8, Canada; 18 McMaster Cochrane Canada and GRADE Centres, McMaster University, Hamilton, ON L8S 4L8, Canada; 19 Center for Immunity, Inflammation and Infectious Diseases, Montreal Clinical Research Institute (IRCM), Montreal, QC H2W 1R7, Canada; emilia.falcone@ircm.qc.ca; 20 Department of Medicine, Université de Montréal, Montreal, QC H3T 1J4, Canada; 21 Division of Nephrology and Hypertension, Department of Internal Medicine, University of Kansas Medical Center, Kansas City, KS 66103, USA

**Keywords:** Post COVID-19, Long COVID, echocardiogram, diagnosis

## Abstract

**Background:** Post-COVID-19 condition (PCC) is a complication following acute COVID-19 infection, which may lead to long-term cardiac abnormalities. This review aimed to assess the prevalence of structural/functional deviations in echocardiography in individuals with PCC compared to patients without PCC. **Methods:** We searched three databases. Two reviewers independently screened articles using LASER Al and extracted relevant data using a piloted Excel sheet. We performed meta-analysis using OpenMeta and RevManWeb and a subgroup analysis based on patients’ settings during acute COVID-19. We assessed the risk of bias using the Hoy et al. tool and the certainty using the Grading of Recommendations Assessment, Development, and Evaluation (GRADE) approach. **Results:** We included 16 studies that reported on differences in echocardiographic findings in patients with or without PCC. Individuals with PCC were more likely to have structural/functional deviations in echocardiographic readings of unclear clinical significance, particularly those who were hospitalized during acute COVID-19. The overall certainty of the evidence was very low due to the high risk of bias, indirectness, and imprecision. **Conclusions:** This review provides insight into the use of echocardiograms and the frequency of test deviations in individuals with PCC. Despite existing evidence, there is a need for future studies to assess the diagnostic test accuracy of echocardiograms in PCC.

## 1. Introduction

After the emergence of SARS-CoV-2, post-COVID-19 condition (PCC) became a significant health concern [1,2]. PCC is a chronic condition characterized by symptoms that persist or emerge at least 12 weeks after infection [3]. Both the terminology and definitions for this condition vary across the literature as commonly used terms include “Long-COVID-19,” “Post-Acute Sequelae of SARS-CoV-2 Infection,” and “Post-Acute COVID-19” [4]. Due to the variability in definitions, this condition is currently identified by evaluating symptoms and ruling out other causes.

It is estimated that 5–40% of the 2.1 million Canadians reporting symptoms associated with PCC may have cardiac or respiratory complaints, regardless of hospitalization during acute COVID-19 infection [5]. The prevalence of breathlessness/dyspnea ranges from 18 to 41%, while cough and chest pain can impact 6.5–18% (95% CI 12–25) and 4.5–15% (95% CI 9–20) of people with PCC, respectively [6,7,8,9]. Moreover, specific cardiac manifestations have also been identified. For example, individuals who survived COVID-19 infection were more likely to develop myocarditis compared to control subjects [10].

Echocardiography is a key investigative tool to assess cardiac function by providing crucial information on cardiac wall thickness, chamber size, wall motion abnormalities, and Pericardial Effusion. Therefore, an echocardiogram may help identify structural or functional cardiac abnormalities that can explain some of the cardiac or respiratory symptoms experienced by adults with PCC, and lead to a change in management, which in turn, can improve symptoms, functional status, and quality of life in adults with PCC. Hence, as part of the Canadian PCC Guidelines (CAN-PCC) diagnostic guidelines development, this systematic review aimed to evaluate the prevalence of structural/functional deviations in echocardiographic findings in individuals with suspected or confirmed PCC to inform guideline recommendations.

## 2. Methods

This review followed the Cochrane Handbook guidance for Systematic Reviews of Interventions. Methodological choices to be ‘rapid’ are identified at specific steps and follow the minimum guidance from the Cochrane Rapid Reviews Methods group [11]. We reported this systematic review according to the Preferred Reporting Items for Systematic reviews and Meta-Analyses (PRISMA) checklist (Supplementary File S1). A protocol for this review was registered in PROSPERO (registration ID: CRD42024526430).

### 2.1. Data Source and Search

We searched MEDLINE (Ovid), Embase, and the Cochrane Central Register of Controlled Trials from December 2019 to March 2024. The search was designed and constructed by experienced CAN-PCC collaborators, including standardized blocks to harmonize systematic reviews for the CAN-PCC project, which was based on a combination of controlled vocabulary (e.g., MeSH) and free text terms. Information specialists used Canada’s Drug Agency Search filters for observational studies and systematic reviews [12,13]. Supplementary File S2 shows the search strategy done for all the PICOs (population, intervention, comparison, and outcomes) included in the guideline for the ease of screening for all PICOs at once.

### 2.2. Eligibility Criteria

Teams of two reviewers screened the title and abstract, and the full text of the studies using LASER AI [14]. A third reviewer resolved conflicts regarding study inclusion or exclusion. Conflicts were addressed using a predefined hierarchy prioritizing the most relevant exclusion reasons. We screened studies for all PICO questions in the guideline simultaneously and categorized included studies according to each PICO.

#### 2.2.1. Study Design

We included randomized controlled trials, cross-sectional, cohort, and case–control studies. We excluded commentaries, editorials, conference abstracts, case reports, narrative reviews, and non-human studies. We included only comparative studies. We excluded systematic reviews but sieved their references for any relevant studies. We included English-language publications only.

#### 2.2.2. Population

For this review, we included adult patients (≥18 years old) who met or were suspected to meet the criteria of the WHO definition and underwent an echocardiogram [15]. Therefore, we excluded studies reporting on patients having symptoms < 3 months after COVID-19 infection, or patients without symptoms. The control population included patients without PCC (either previously healthy or those who recovered from COVID-19 without developing PCC) who underwent the test (for prevalence).

#### 2.2.3. Setting

We included studies reporting on patients in any setting (hospitalized, non-hospitalized, mixed).

#### 2.2.4. Outcomes

We included values for each of the tests that were prioritized by the panel based on patient-important outcomes.

Based on a discussion with experts in the field from the panel, the following findings were sought: Left Ventricular Ejection Fraction (LVEF), Left Ventricular Hypertrophy (LVH), Left Ventricular End Diastolic Volume (LVEDV), Tricuspid Annular Plane Systolic Excursion (TAPSE), Right Ventricular Systolic Pressure (RVSP), Right Atrial Diameter, Right Ventricular Diameter, Pericardial Effusion, and Abnormal Echocardiogram.

### 2.3. Data Extraction

We extracted data using a piloted and standardized form that included study characteristics in Excel. The form included general study details, risk of bias assessment, population details and relevant details per outcome (e.g., definition, measurement, duration of follow-up). Extractions were performed independently by two reviewers. Discrepancies were resolved by discussion, or if needed, by a third reviewer.

### 2.4. Statistical Analysis

For meta-analysis of diagnostic test accuracy, we only included studies reporting on sensitivity and specificity. As for the meta-analysis of prevalence studies, for continuous outcomes, we used RevMan Web to pool data for more than two studies (i.e., mean difference with 95% CI). For dichotomous data, we used RevMan Web to pool comparative data (we used odds ratio (OR) with 95% CI). We used fixed-effect meta-analysis if the outcome included 2 studies because estimation of variance between very few studies in the random-effect model would be unstable, and random-effect meta-analysis if the outcome included >2 studies. We did a subgroup analysis for the following groups: hospitalized, non-hospitalized, and mixed populations. We used forest plots to display all comparative data synthesized.

### 2.5. Quality Assessment

#### 2.5.1. Risk of Bias

We assessed the risk of bias using the tool developed by Hoy et al. [16]. Two reviewers (J.K. and M.A.) performed the assessment.

#### 2.5.2. Certainty of Evidence

We assessed the quality of evidence using the Grading of Recommendations Assessments, Development, and Evaluation (GRADE) approach. Two reviewers (J.K. and M.A.) familiar with GRADEPro GDT software (https://gradepro.org) (accessed on 1 March 2024) constructed a summary of findings table for each research question. We considered study design, risk of bias, inconsistency, indirectness, imprecision, and publication bias for the assessment of the quality of evidence for each outcome [17,18].

## 3. Results

Our main finding was the lack of studies to accurately evaluate the diagnostic utility of echocardiography in patients with PCC, regardless of their hospitalization status during acute infection. Therefore, all evidence found was on the prevalence of a positive test in individuals with PCC compared to those without PCC. A total of 16 non-randomized studies were included in the quantitative synthesis after screening. The PRISMA flow diagram is shown in detail in Supplementary File S3. Key study characteristics, including population demographics and outcome measures, are summarized in Table 1 [19,20,21,22,23,24,25,26,27,28,29,30,31,32,33,34].

### 3.1. Left Ventricular Ejection Fraction (LVEF) and Left Ventricular End Diastolic Volume (LVEDV)

- Continuous data: A total of 11 studies (*n* = 1764) reported differences in LVEF between patients with PCC and patients without PCC in hospitalized (*n* = 1028), non-hospitalized (*n* = 488), and mixed settings (*n* = 248). The pooled estimates for absolute mean difference (95% CI) in LVEF between patients with PCC and patients without PCC were −1.23% [95% CI (−1.92%, −0.54%)], −0.5% [95% CI (−0.55%, −0.45%)], −1.15% [95% CI (−1.81%, −0.49%)] in hospitalized, non-hospitalized, and mixed settings, respectively. Two studies (*n* = 196) reported a pooled estimate for the absolute mean difference in LVEDV between patients with PCC and patients without PCC in hospitalized patients as −1.49% [95% CI (−3.92%, 0.94%)] (Supplementary File S4, Figure S1a–d).
- Dichotomous data (LVEF < 50%): One study (*n* = 83) reported a pooled odds ratio (95% CI) for LVEF < 50% between patients with PCC and patients without PCC of 1.72 [95% CI (0.45, 6.61)] in hospitalized patients (Supplementary File S4, Figure S1e).

Tricuspid Annular Plane Systolic Excursion (TAPSE):

- Continuous data: Exactly 9 studies (*n* = 1252) reported differences in TAPSE between patients with PCC and patients without PCC in hospitalized (*n* = 1010) and mixed settings (*n* = 242). The pooled estimates for mean difference (95% CI) in TAPSE between patients with PCC and patients without PCC were −0.46% [95% CI (−1.50%, 0.57%)] and −0.11% [95% CI (−0.88%, 0.67%)] in hospitalized and mixed settings, respectively (Supplementary File S4, Figure S2a,b).

### 3.2. Right Atrial Diameter (RAD), Right Ventricular Systolic Pressure (RVSP), and Right Ventricular Diameter (RVD)

- Continuous data: One study (*n* = 202) reported the pooled estimate for mean difference in RAD between patients with PCC and patients without PCC in hospitalized patients as 2.11% [95% CI (0.86%, 3.34%)]. One study (*n* = 70) reported the pooled estimate for mean difference in RVSP between patients with PCC and patients without PCC in hospitalized patients as 5.25% [95% CI (4.4%, 6.1%)] (Supplementary File S4, Figure S3b). Two studies (*n* = 634) reported differences in RVD between patients with PCC and patients without PCC in hospitalized (*n* = 146) and non-hospitalized (*n* = 488) patients. The pooled estimates for mean difference (95% CI) in RVD between patients with PCC and patients without PCC were 0.77% [95% CI (−0.98%, 2.38%)] and 0.00% [95% CI (−0.53%, 0.53%)] in hospitalized and non-hospitalized patients, respectively (Supplementary File S4, Figure S3a–d).

In one study (*n* = 102) the risk difference in pleural effusion between patients with PCC and patients without PCC was 0.09 (95% CI 0 to 0.17) in patients from mixed settings. In one study involving 40 patients with PCC and 36 patients without PCC, no abnormal echocardiographic findings were observed in either group (Supplementary File S4).

The certainty of evidence was assessed as very low due to serious concerns of risk of bias (confounding with no adjustments), indirectness of the comparison (patients with PCC versus patients without PCC) instead of the intended comparison (test vs. no test), small sample sizes, and the use of non-comparative data in the case of the absence of comparative data. Table 2 provides a comprehensive summary of the findings, with their certainty assessment. All corresponding forest plots are included in Supplementary File S4 for pooled outcomes.

## 4. Discussion

This systematic review informed the CAN-PCC diagnostic guideline recommendations. Although the primary objective was to evaluate the impact of testing versus not testing individuals suspected of having PCC, the absence of validated reference tests for PCC led the review to focus on prevalence studies. These studies compared test outcomes between individuals with PCC and those without, regardless of their history of SARS-CoV-2 infection. The analysis was based on setting, as recommended by the panel’s expert input, as they expected that there would be published research regarding potential differences between hospitalized and non-hospitalized patients. Therefore, this review primarily provides descriptive echocardiographic findings rather than evaluating the effectiveness of echocardiography as a diagnostic tool.

### 4.1. Interpretation of the Results

While the panel considered the decision threshold to be the line of no effect when evaluating imprecision, we recognize that some statistically significant changes may have been not clinically meaningful. Therefore, several findings while reaching statistical significance, were viewed by the guideline panel as clinically not that meaningful. Lower LVEF was shown in individuals with PCC compared to individuals without PCC, especially those previously hospitalized. However, no statistically significant differences were observed in LVED or TAPSE between those with or without PCC. Previously hospitalized individuals with PCC exhibited greater RAD and RVSP but not RVD compared to those without PCC. These results are consistent with a prior study that reported structural/functional deviations in echocardiography such as myocardial edema and fibrosis in individuals with PCC [35]. In addition, one other study found that individuals who recovered from COVID-19 (even without developing PCC) had higher risk of myocarditis than individuals who did not have COVID-19 [10].

### 4.2. Limitations

One limitation of this review is that, despite including studies that used the WHO definition, there was heterogeneity in the follow-up period between some studies (more than 3, 6 or 12 months). Despite this variability, LVEF remained consistent among hospitalized patients. Another crucial limitation is that we are not commenting on the clinically meaningful differences, but rather on the statistical significance when assessing the certainty of evidence—due to the nature of the studies and the lack of reporting of dichotomous data. Therefore, conclusions made about the clinical use of echocardiography in this population should be interpreted with caution. Importantly, this study reviewed associative data and cannot provide inferences on causation. In other words, it is not clear whether those with pre-existing echocardiography abnormalities are predisposed to developing PCC, or whether those with PCC developed structural/functional deviations in echocardiography post-COVID, since the studies reviewed do not provide pre-COVID baseline data. Thus, while it might be tempting to conclude that COVID or PCC can cause cardiac abnormalities, our results do not support such a conclusion. In addition, it is also important to acknowledge that this review was mostly based on very low certainty evidence due to limited data on diagnostic accuracy and the observational nature of the studies. This could be explained by the absence of reference standard testing to diagnose PCC. It is crucial to consider that performing echocardiography in individuals with suspected or confirmed PCC could reflect previously undiagnosed structural/functional abnormalities. However, this cannot be determined without a baseline echocardiogram obtained before infection. Another limitation is that outcomes with pooled studies included comparators of either previously healthy patients or patients who had COVID-19 but did not develop PCC, and, while we acknowledge that pooling them may introduce bias, a stratified analysis was not feasible due to insufficient data. Moreover, subgroup analysis comparing hospitalized and non-hospitalized populations should be interpreted cautiously, as the small number of studies contributing to each group limited the use of formal statistical tests for interaction. Finally, while our data demonstrates some echocardiographic differences between individuals with or without PCC, the clinical relevance of these sometimes minute, though statistically significant, differences remain unclear. It is also unclear whether these changes are temporary (i.e., seen only in cross-section at the time of the study) or persistent, as no studies provided longitudinal assessments with echocardiography.

### 4.3. Implications for Future Practice and Research

While other studies reported on cardiovascular manifestations in PCC, they either did not report them based on echocardiogram [36] or did not report them as pooled outcomes via meta-analysis, or used other definitions, including those with symptoms ≥ 4 weeks [35]. Some have also focused on one etiology in cardiovascular involvement [10]. To our knowledge, this is the first systematic review specifically addressing the use of echocardiography in individuals with PCC. Our review has focused on the overall cardiac manifestations tested by echocardiogram in individuals with suspected or confirmed PCC. This provides an overall perspective of what an echocardiogram can detect and how it can do so in this specific population. Moreover, in patients with PCC and suspected myocardial injury, echocardiography may help assess for intracardiac thrombi, and thus, a standardized approach to cardiac masses may help guide further imaging if needed [37].

## 5. Conclusions

There is still a gap in the current literature in diagnostic accuracy studies evaluating echocardiography against a reference standard in PCC and comparative studies between testing and no testing, which poses an ongoing challenge to determining the clinical utility of echocardiography in individuals with PCC. Consequently, further research is warranted to address these gaps by using baseline and longitudinal echocardiographic assessments, adjustment for confounders (such as comorbidities like hypertension and obesity), and inclusion of clinically meaningful endpoints, including thresholds for MCID.

## Figures and Tables

**Table 1:** Characteristics of studies included.

Author Year	Country	Study Design	Population Characteristics	Comparator (If Applicable)	Definition of PCC	Test Performed	Outcomes Reported
Aparisi 2021 [19]	Spain	Prospective study	Hospitalized and non-hospitalized (*n* = 41)	Patients who had COVID-19 but did not develop PCC (*n* = 29)	≥3 months	Transthoracic Echocardiogram	LVEF TAPSE
Acanfora 2022 [20]	Italy	Observational cohort study	Hospitalized (*n* = 30)	Patients who never tested positive for PCC (*n* = 20)	≥3 months	Echocardiogram (Type not specified)	LVEDV LVEF
Aranyó 2022 [21]	Spain	Prospective observational	Hospitalized and non-hospitalized (*n* = 200)	N/A	≥3 months	Echocardiogram	Abnormal Echocardiogram
Aparisi 2022 [22]	Spain	Prospective cohort study study	Hospitalized and non-hospitalized (*n* = 10)	Patients who had COVID-19 but did not develop PCC (*n* = 60)	≥3 months	Transthoracic Echocardiogram	TAPSE
Beaudry 2022 [23]	Canada	Cross-sectional study	Non-hospitalized (*n* = 28)	Patients who had COVID-19 but did not develop PCC (*n* = 14)	≥3 months	Transthoracic Echocardiogram	LVEF LVEDV TAPSE RVSP
Chudzik 2022 [24]	Poland	Prospective observational	Non-hospitalized (*n* = 218)	Patients who had COVID-19 but did not develop PCC (*n* = 270)	≥3 months	Echocardiogram	LVEF TAPSE
Duran 2022 [25]	Turkey	Case–control study	Hospitalized (*n* = 102)	Patients who never tested positive for COVID-19 (*n* = 100)	≥12 months	Transthoracic Echocardiogram	LVEDD LVEF RA diameter TAPSE
Durstenfeld 2022 [26]	USA	Prospective cohort study	Hospitalized and non-hospitalized (*n* = 47)	Patients who had COVID-19 but did not develop PCC (*n* = 55)	≥3 months	Transthoracic Echocardiogram	LVEF TAPSE RVSV
Jutant 2022 [27]	France	Prospective cohort	Hospitalized (*n* = 40)	Patients who had COVID-19 but did not develop PCC (*n* = 43)	≥3 months	Transthoracic Echocardiogram	LVEF
Luchian 2022 [28]	Belgium	Prospective study	Hospitalized (*n* = 23)	Patients who had COVID-19 but did not develop PCC (*n* = 43)	≥ 12 months	Transthoracic Echocardiogram	LVEF TAPSE
Cecchetto 2023 [29]	Italy	Retrospective cohort study	Hospitalized (*n* = 115)	Patients who had COVID-19 but did not develop PCC (*n* = 110)	≥3 months	Transthoracic Echocardiogram	LVEF TAPSE
Cecchetto 2023 [30]	Italy	Retrospective cohort study	Hospitalized (*n* = 144)	Patients who had COVID-19 but did not develop PCC (*n* = 81)	≥3 months	Transthoracic Echocardiogram	TAPSE
Gryglewska-Wawrzak 2023 [31]	Poland	Prospective cohort study	Hospitalized patients (*n* = 44)	Patients who had COVID-19 but did not develop PCC (*n* = 102)	3–6 months	Echocardiogram (Type not reported)	LVEF EDV TAPSE
Niebauer 2023 [32]	Austria	Prospective study	Hospitalized (*n* = 113)	Patients who had COVID-19 but did not develop PCC (*n* = 37)	≥6 months	Transthoracic Echocardiogram	LVEF
Tas 2023 [33]	Turkey	Prospective cohort study	Hospitalized (*n* = 51)	Patients who never tested positive for COVID-19 (*n* = 95)	≥6 months	Transthoracic Echocardiogram	LVEF TAPSE
Rajotiya 2024 [34]	India	Prospective case–control study	Hospitalized (*n* = 23)	Patients who had COVID-19 but did not develop PCC (*n* = 20)	≥3 months	Transthoracic Echocardiogram	LVEF

N/A: Not applicable; LVEF: Left ventricular ejection fraction; TAPSE: Tricuspid Annular Plane Systolic Excursion; LVEDV: Left ventricular end diastolic volume; RVSP: Right Ventricular Systolic Pressure; RA: Right atrium.

**Table 2:** GRADE summary of findings for the comparison of echocardiograms in patients with PCC versus echocardiograms in patients without PCC.

Certainty Assessment							№ of Patients		Effect		Certainty
№ of Studies	Study Design	Risk of Bias	Inconsistency	Indirectness	Imprecision	Other Considerations	Patients with PCC	Patients Without PCC	Relative (95% CI)	Absolute (95% CI)	
Left Ventricular Ejection Fraction-Hospitalized											
8	non-randomized studies	serious ^a^	not serious	serious ^b^	not serious	none	501	527	-	MD 1.23% lower (1.92 lower to 0.54 lower)	⨁◯◯◯ Very low ^a,b^
Left Ventricular Ejection Fraction-Non-Hospitalized											
1	non-randomized studies	serious ^a^	not serious	serious ^b^	not serious	none	218	270	-	MD 0.5% lower (0.55 lower to 0.45 lower)	⨁◯◯◯ Very low ^a,b^
Left Ventricular Ejection Fraction-Mixed											
2	non-randomized studies	serious ^a^	not serious	serious ^b^	serious ^c^	none	91	157	-	MD 1.15% lower (1.81 lower to 0.49 lower)	⨁◯◯◯ Very low ^a,b,c^
Left Ventricular Ejection Fraction < 50%-Hospitalized											
1	non-randomized studies	serious ^a^	not serious	serious ^b^	serious ^c^	none	6/40 (15.0%)	4/43 (9.3%)	OR 1.72 (0.45 to 6.61)	57 more per 1000 (from 49 fewer to 311 more)	⨁◯◯◯ Very low ^a,b,c^
Left Ventricular End Diastolic Volume-Hospitalized											
2	non-randomized studies	serious ^a^	not serious	serious ^b^	serious ^c,d^	none	74	122	-	MD 1.49 mL lower (3.92 lower to 0.94 higher)	⨁◯◯◯ Very low ^a,b,c,d^
Tricuspid Annular Plane Systolic Excursion (TAPSE)-Hospitalized											
6	non-randomized studies	serious ^a^	not serious	serious ^b^	serious ^d^	none	479	531	-	MD 0.46 mL lower (1.5 lower to 0.57 higher)	⨁◯◯◯ Very low ^a,b,d^
Tricuspid Annular Plane Systolic Excursion (TAPSE)-Mixed											
3	non-randomized studies	serious ^a^	not serious	serious ^b^	serious ^c,d^	none	98	144	-	MD 0.11 mL lower (0.88 lower to 0.67 higher)	⨁◯◯◯ Very low ^a,b,c,d^
Right Atrial Diameter-Hospitalized											
1	non-randomized studies	serious ^a^	not serious	serious ^b^	serious ^c^	none	102	100	-	MD 2.1 mm higher (0.86 higher to 3.34 higher)	⨁◯◯◯ Very low ^a,b,c^
Right Ventricular Systolic Pressure-Mixed											
1	non-randomized studies	serious ^a^	not serious	serious ^b^	very serious ^c^	none	41	29	-	MD 5.25 mmHg higher (4.4 higher to 6.1 higher)	⨁◯◯◯ Very low ^a,b,c^
Right Ventricular Diameter-Hospitalized											
1	non-randomized studies	serious ^a^	not serious	serious ^b^	very serious ^c^	none	51	95	-	MD 0.7 mm higher (0.98 lower to 2.38 higher)	⨁◯◯◯ Very low ^a,b,c^
Right Ventricular Diameter-Non-Hospitalized											
1	non-randomized studies	serious ^a^	not serious	serious ^b^	very serious ^d^	none	218	270	-	MD 0 mm (0.53 lower to 0.53 higher)	⨁◯◯◯ Very low ^a,b,d^
Pericardial Effusion-Mixed											
1	non-randomized studies	serious ^a^	not serious	serious ^b^	very serious ^c^	none	4/47 (8.5%)	0/55 (0.0%)	Risk Difference 0.09 (0 to 0.17)	0 fewer per 1000 (from 0 fewer to 0 fewer)	⨁◯◯◯ Very low ^a,b,c^
Abnormal Echocardiogram-Mixed											
1	non-randomized studies	serious ^a^	not serious	serious ^b^	very serious ^c^	none	In one study involving 40 patients with PCC and 36 patients without PCC, no Abnormal Echocardiograms were found in either group.				⨁◯◯◯ Very low ^a,b,c^

CI: confidence interval; MD: mean difference; OR: odds ratio ^a^. Risk of bias is serious due to the possible confounding effect caused by the baseline characteristics of the samples that were not adjusted.; ^b^. Downgraded for indirectness because the studies are assessing a comparison between post-COVID-19 condition patients vs. non-post-COVID-19 condition patients which is not the comparison of interest (ECHO vs. no ECHO). ^c^. Small number of events and patients included.; ^d^. Confidence interval crosses line of no effect. ⨁◯◯◯ Very low certainty.

## Data Availability

The original contributions presented in this study are included in the article/Supplementary Materials. Further inquiries can be directed to Reem A. Mustafa.

## Supplementary Materials

The following supporting information can be downloaded at: https://www.mdpi.com/article/10.3390/jcm15124643/s1, Supplementary File S1: PRISMA 2020 Checklist; Supplementary File S2: Search Strategy of all PICOs included in the guideline; Supplementary File S3: PRISMA flow chart; Supplementary File S4: Forest Plots.
