# Supplementary material for: Echocardiogram Testing in Patients with Post-COVID-19 Condition: A Systematic Review and Meta-Analysis
Source: J Clin Med. 2026 Jun 15;15(12):4643. doi: 10.3390/jcm15124643 (PMC13301671; doi:10.3390/jcm15124643)

Supplementary File S1: *PRISMA 2020 Checklist*

Section and Topic	Item #	Checklist item	Location where item is reported
**TITLE**			
Title	1	Identify the report as a systematic review.	Page 1
**ABSTRACT**			
Abstract	2	See the PRISMA 2020 for Abstracts checklist.	Page 3
**INTRODUCTION**			
Rationale	3	Describe the rationale for the review in the context of existing knowledge.	Page 4
Objectives	4	Provide an explicit statement of the objective(s) or question(s) the review addresses.	Page 4
**METHODS**			
Eligibility criteria	5	Specify the inclusion and exclusion criteria for the review and how studies were grouped for the syntheses.	Page 5-6
Information sources	6	Specify all databases, registers, websites, organisations, reference lists and other sources searched or consulted to identify studies. Specify the date when each source was last searched or consulted.	Page 5
Search strategy	7	Present the full search strategies for all databases, registers and websites, including any filters and limits used.	Supplementary file 1
Selection process	8	Specify the methods used to decide whether a study met the inclusion criteria of the review, including how many reviewers screened each record and each report retrieved, whether they worked independently, and if applicable, details of automation tools used in the process.	Page 5
Data collection process	9	Specify the methods used to collect data from reports, including how many reviewers collected data from each report, whether they worked independently, any processes for obtaining or confirming data from study investigators, and if applicable, details of automation tools used in the process.	Page 5-6
Data items	10a	List and define all outcomes for which data were sought. Specify whether all results that were compatible with each outcome domain in each study were sought (e.g. for all measures, time points, analyses), and if not, the methods used to decide which results to collect.	Page 5
	10b	List and define all other variables for which data were sought (e.g. participant and intervention characteristics, funding sources). Describe any assumptions made about any missing or unclear information.	Page 5
Study risk of bias assessment	11	Specify the methods used to assess risk of bias in the included studies, including details of the tool(s) used, how many reviewers assessed each study and whether they worked independently, and if applicable, details of automation tools used in the process.	Page 6
Effect measures	12	Specify for each outcome the effect measure(s) (e.g. risk ratio, mean difference) used in the synthesis or presentation of results.	Page 6
Synthesis methods	13a	Describe the processes used to decide which studies were eligible for each synthesis (e.g. tabulating the study intervention characteristics and comparing against the planned groups for each synthesis (item #5)).	Page 6
	13b	Describe any methods required to prepare the data for presentation or synthesis, such as handling of missing summary statistics, or data conversions.	Page 6
	13c	Describe any methods used to tabulate or visually display results of individual studies and syntheses.	Page 6
	13d	Describe any methods used to synthesize results and provide a rationale for the choice(s). If meta-analysis was performed, describe the model(s), method(s) to identify the presence and extent of statistical heterogeneity, and software package(s) used.	Page 6
	13e	Describe any methods used to explore possible causes of heterogeneity among study results (e.g. subgroup analysis, meta-regression).	Page 6
	13f	Describe any sensitivity analyses conducted to assess robustness of the synthesized results.	Page 6
Reporting bias assessment	14	Describe any methods used to assess risk of bias due to missing results in a synthesis (arising from reporting biases).	Page 6

Supplementary File S1: *PRISMA 2020 Checklist*

Section and Topic	Item #	Checklist item	Location where item is reported
Certainty assessment	15	Describe any methods used to assess certainty (or confidence) in the body of evidence for an outcome.	Page 6
**RESULTS**			
Study selection	16a	Describe the results of the search and selection process, from the number of records identified in the search to the number of studies included in the review, ideally using a flow diagram.	Supplementary file 2
	16b	Cite studies that might appear to meet the inclusion criteria, but which were excluded, and explain why they were excluded.	Supplementary file 2
Study characteristics	17	Cite each included study and present its characteristics.	Table 1
Risk of bias in studies	18	Present assessments of risk of bias for each included study.	Tables 2 and 3
Results of individual studies	19	For all outcomes, present, for each study: (a) summary statistics for each group (where appropriate) and (b) an effect estimate and its precision (e.g. confidence/credible interval), ideally using structured tables or plots.	Tables 2 and 3
Results of syntheses	20a	For each synthesis, briefly summarise the characteristics and risk of bias among contributing studies.	Tables 2 and 3
	20b	Present results of all statistical syntheses conducted. If meta-analysis was done, present for each the summary estimate and its precision (e.g. confidence/credible interval) and measures of statistical heterogeneity. If comparing groups, describe the direction of the effect.	Supplementary file 3
	20c	Present results of all investigations of possible causes of heterogeneity among study results.	Pages 7-8
	20d	Present results of all sensitivity analyses conducted to assess the robustness of the synthesized results.	Tables 2 and 3
Reporting biases	21	Present assessments of risk of bias due to missing results (arising from reporting biases) for each synthesis assessed.	Tables 2 and 3
Certainty of evidence	22	Present assessments of certainty (or confidence) in the body of evidence for each outcome assessed.	Tables 2 and 3
**DISCUSSION**			
Discussion	23a	Provide a general interpretation of the results in the context of other evidence.	Page 10
	23b	Discuss any limitations of the evidence included in the review.	Page 10-11
	23c	Discuss any limitations of the review processes used.	Page 10-11
	23d	Discuss implications of the results for practice, policy, and future research.	Page 10-11
**OTHER INFORMATION**			
Registration and protocol	24a	Provide registration information for the review, including register name and registration number, or state that the review was not registered.	Page 5
	24b	Indicate where the review protocol can be accessed, or state that a protocol was not prepared.	Page 5
	24c	Describe and explain any amendments to information provided at registration or in the protocol.	Page 5
Support	25	Describe sources of financial or non-financial support for the review, and the role of the funders or sponsors in the review.	Page 13
Competing interests	26	Declare any competing interests of review authors.	Page 14
Availability of data, code and	27	Report which of the following are publicly available and where they can be found: template data collection forms; data extracted from included studies; data used for all analyses; analytic code; any other materials used in the review.	Page 15

Supplementary File S1: *PRISMA 2020 Checklist*

Section and Topic	Item #	Checklist item	Location where item is reported
other materials			

Supplementary File S2: *Search Strategy of all PICOs included in the guideline*

We did the search strategy in 4 batches due to the rapid nature of this review. Each batch included one or more PICO question(s) of this guideline. After including all studies, duplicates were removed.

<u>Search Strategy Batch #1:</u>

1)In people with suspected PCC who have fatigue or dizziness, should we use validated tests to assess functional status (e.g., Patient-reported outcomes measurement information system; post-covid-19 functional status scale, and EuroQol-5D (EQ-5D) versus not use these validated tests to assess functional status?

MEDLINE

Database: Ovid MEDLINE(R) ALL <1946 to February 26, 2024>

Search Date: 27 February 2024

1 patient reported outcome measures/ (14619)

2 (patient reported adj3 outcome*).tw,kf,kw. (42506)

3 functional status/ and (assess* or instrument* or measur* or questionnaire* or scale* or score* or scoring or test* or tool* or validat*).tw,kf,kw. (1216)

4 (functional adj2 (scale* or status*)).tw,kf,kw. (41727)

5 (EuroQol-5D* or EuroQol5d* or eq-5d* or eq5d*).tw,kf,kw. (15304)

6 (fatigue severity scale* or facit-f*).tw,kf,kw. (2558)

7 "long covid symptom and impact tool*".tw,kf,kw. (1)

8 ((post-acute or long) adj2 covid* adj2 "quality of life").tw,kf,kw. (28)

9 (PAC-19QoL* or PAC19QoL* or PAC-19-QoL*).tw,kf,kw. (5)

10 (Short-Form-36* or short-form36* or shortform-36* or shortform36*).tw,kf,kw. (12324)

11 (sf-36* or sf36*).tw,kf,kw. (27420)

12 or/1-11 [tools and measures] (129400)

13 Post-Acute COVID-19 Syndrome/ (3081)

14 ((chronic* or late sequela* or prolong*) adj2 (COVID or COVID-19 or COVID19 or coronavirus* or corona virus* or 2019-nCoV or 19nCoV or 2019nCoV or nCoV or n-CoV or "CoV 2" or CoV2 or SARS-CoV-2 or SARS-CoV2 or SARSCoV-2 or SARSCoV2 or SARS2 or SARS-2 or severe acute respiratory syndrome coronavirus 2 or 2019-novel CoV or Sars-coronavirus2 or Sars-coronavirus-2 or SARS-like coronavirus* or novel coronavirus* or novel corona virus* or novel CoV or OC43 or NL63 or 229E or HKU1 or HCoV* or Sars-coronavirus*)).tw,kw,kf. (2740)

15 ((long or longterm* or long-term* or "long haul" or "long haulers" or "long hauler" or long haul*) adj (COVID or COVID-19 or COVID19 or coronavirus* or corona virus* or 2019-nCoV or 19nCoV or 2019nCoV or nCoV or n-CoV or "CoV 2" or CoV2 or SARS-CoV-2 or SARS-CoV2 or

SARSCoV-2 or SARSCoV2 or SARS2 or SARS-2 or severe acute respiratory syndrome coronavirus 2 or 2019-novel CoV or Sars-coronavirus2 or Sars-coronavirus-2 or SARS-like coronavirus* or novel coronavirus* or novel corona virus* or novel CoV or OC43 or NL63 or 229E or HKU1 or HCoV* or Sars-coronavirus*)).tw,kw,kf. (5703)

16 ((after* or following or post* or "post acute") adj (COVID or COVID-19 or COVID19 or coronavirus* or corona virus* or 2019-nCoV or 19nCoV or 2019nCoV or nCoV or n-CoV or "CoV 2" or CoV2 or SARS-CoV-2 or SARS-CoV2 or SARSCoV-2 or SARSCoV2 or SARS2 or SARS-2 or severe acute respiratory syndrome coronavirus 2 or 2019-novel CoV or Sars-coronavirus2 or Sars-coronavirus-2 or SARS-like coronavirus* or novel coronavirus* or novel corona virus* or novel CoV or OC43 or NL63 or 229E or HKU1 or HCoV* or Sars-coronavirus*) adj3 (chronic* or clinical feature? or comorbid* or "co-morbid*" or complication* or condition* or consequence* or convalescen* or discharge* or disease* or disorder* or effect? or fatigue or followup* or follow-up* or function* or illness* or impact* or infection* or issue* or multimorbid* or "multi morbid*" or nonrecover* or non-recover* or persist* or prognos* or prolonged or recover* or recuperat* or recurr* or rehabilitat* or relaps* or residual or risk* or secondary or sequela* or sickness* or sign or signs or survivor* or survival* or syndrome* or symptom*)).tw,kw,kf. (8564)

17 or/13-16 [PCC -Long Covid Set1] (15327)

18 exp Covid-19/ (256471)

19 SARS-CoV-2/ (165856)

20 SARS-CoV-2 variants.mp. (10747)

21 (COVID-19 or COVID19).tw,kw,kf. (362748)

22 (((coronavirus* or corona virus*) and (hubei or wuhan or beijing or shanghai)) or (wuhan adj5 virus*) or (2019-nCoV or 19nCoV or 2019nCoV) or (nCoV or n-CoV or "CoV 2" or CoV2)).tw,kw,kf. (142151)

23 (SARS-CoV-2 or SARS-CoV2 or SARSCoV-2 or SARSCoV2 or SARS2 or SARS-2 or severe acute respiratory syndrome coronavirus 2).tw,kw,kf. (141503)

24 (2019-novel CoV or Sars-coronavirus2 or Sars-coronavirus-2 or SARS-like coronavirus* or ((novel or new or nouveau) adj2 (CoV or nCoV or covid or coronavirus* or corona virus or Pandemi*2)) or (coronavirus* and pneumonia)).tw,kw,kf. (31253)

25 (novel coronavirus* or novel corona virus* or novel CoV).tw,kw,kf. (14072)

26 (((coronavirus* or corona virus*) adj2 "2019") or ((coronavirus* or corona virus*) adj2 "19") or (coronavirus 2 or corona virus 2)).tw,kw,kf. (97002)

27 (OC43 or NL63 or 229E or HKU1 or HCoV* or Sars-coronavirus*).tw,kw,kf. (5167)

28 COVID-19.rx,px,ox. or severe acute respiratory syndrome coronavirus 2.os. (2299)

29 (coronavirus* or corona virus* or COVID).ti. (292725)

30 or/18-29 [COVID-19] (420261)

31 ((long* or persist* or prolonged or residual) adj8 ((olfactory or chemosensor* or chemo

sensor*) adj (disorder* or dysfunction*))).tw,kw,kf. (239)
32 (((post acute or postacute or sub-acute or subacute or chronic) adj sequela*) or PASC or PACS).tw,kw,kf. (7813)
33 (post* adj3 (care or aftercare* or rehabilitati*) adj2 (clinic or clinics or centre or center or centres or centers or program*)).tw,kw,kf. (1417)
34 ((post-intensive care or postintensive care or post-ICU) adj syndrome*).tw,kw,kf. (761)
35 (recovery adj2 (clinic or clinics or centre or center or centres or centers or program*)).tw,kw,kf. (2567)
36 ((chronic* or continuous* or continual* or continuing* or delay* or endur* or extend* or f luctuat* or gradual* or lasting* or legacy* or lengthy* or linger* or long* or "medium* term*" or mediumterm* or multisystem* or "multi system*" or ongoing* or permanent* or persist* or prolong* or protract* or relaps* or remission* or remit* or residual* or slow* or subacute* or "sub acute*") adj3 recover*).tw,kw,kf. (39935)
37 ((after discharg* or following discharg* or postacute* or "post acute*" or "post-acute" or postdischarg* or "post discharge" or "post discharging" or posthospital* or post-hospital* or postinfect* or "post infection" or "post infective*" or postviral* or "post viral*" or postvirus* or "post virus*" or postcritical or post-critical or postintensive or post-intensive or post-ICU) adj3 recover*).tw,kw,kf. (782)
38 ((chronic* or continuous* or continual* or continuing* or delay* or endur* or extend* or f
luctuat* or gradual* or lasting* or legacy* or lengthy* or linger* or long* or "medium* term*" or mediumterm* or multisystem* or "multi system*" or ongoing or permanent* or persist* or prolong* or protract* or relaps* or remission* or remit* or residual* or slow* or subacute* or "sub acute*") adj3 (aftercare* or after-care* or complication? or condition* or consequence? or convalescen* or disabilit* or disease* or disorder* or feature* or illness* or issue* or prognos* or sequela* or sign or signs or suffering? or symptom* or syndrome* or recuperat*)).tw,kw,kf. (931643)
39 ((after discharg* or following discharg* or late-onset or postacute* or "post acute*" or postdischarg* or "post discharge" or "post discharging" or posthospital* or post-hospital* or postinfect* or "post infection" or "post infective*" or postviral* or "post viral*" or postvirus* or "post virus*" or postcritical or post-critical or postintensive or post-intensive or post-ICU or survivor* or survived) adj3 (complication? or condition* or consequence? or convalescen* or disabilit* or disease* or disorder* or feature* or illness* or issue* or prognos* or sequela* or sign or signs or suffering? or symptom* or syndrome* or recuperat*)).tw,kw,kf. (27883)
40 (nonrecover* or "non recover*" or "not recover*" or ("long* haul*" or long haul* or longhaul* or "long* tail*" or longtail* or longduration* or "long duration*" or longlast* or "long last*" or longstanding* or "long standing*" or "medium* term*" or mediumterm*)).tw,kw,kf.

(144614)
41 or/31-40 [Long haul terms] (1124865)
42 30 and 41 [COVID-19 AND Long Haul: Part 2 of PCC Strategy] (30792)
43 Long Term Adverse Effects/ and 30 [Part 3 of PCC sensitive strategy] (12)
44 exp animals/ not exp humans/ (5199687)
45 (2019* or 202*).yr. (7712463)
46 ((or/17,42-43) and 45) not 44 [PCC/Long Covid Sensitive-ML] (38426)
47 12 and 46 (792)
48 limit 47 to english language (765)

EMBASE

Database: Embase <1996 to 2024 February 26>
Search Date: 27 February 2024

1 patient reported outcome/ (58579)
2 (patient reported adj3 outcome*).tw,kf,kw. (67789)
3 functional status/ and (assess* or instrument* or measur* or questionnaire* or scale* or score* or scoring or test* or tool* or validat*).tw,kf,kw. (54568)
4 (functional adj2 (scale* or status*)).tw,kf,kw. (59804)
5 (EuroQol-5D* or EuroQol5d* or eq-5d* or eq5d*).tw,kf,kw. (28676)
6 (fatigue severity scale* or facit-f*).tw,kf,kw. (5868)
7 "long covid symptom and impact tool*".tw,kf,kw. (1)
8 ((post-acute or long) adj2 covid* adj2 "quality of life").tw,kf,kw. (33)
9 (PAC-19QoL* or PAC19QoL* or PAC-19-QoL*).tw,kf,kw. (5)
10 (Short-Form-36* or short-form36* or shortform-36* or shortform36*).tw,kf,kw. (16934)
11 (sf-36* or sf36*).tw,kf,kw. (46831)
12 or/1-11 (241996)
13 long COVID/ (6568)
14 ((chronic* or late sequela* or prolong*) adj2 (COVID or COVID-19 or COVID19 or coronavirus* or corona virus* or 2019-nCoV or 19nCoV or 2019nCoV or nCoV or n-CoV or "CoV 2" or CoV2 or SARS-CoV-2 or SARS-CoV2 or SARSCoV-2 or SARSCoV2 or SARS2 or SARS-2 or severe acute respiratory syndrome coronavirus 2 or 2019-novel CoV or Sars-coronavirus2 or Sars-coronavirus-2 or SARS-like coronavirus* or novel coronavirus* or novel corona virus* or novel CoV or OC43 or NL63 or 229E or HKU1 or HCoV* or Sars-coronavirus*)).tw,kw,kf. (2960)
15 ((long or longterm* or long-term* or "long haul" or "long haulers" or "long hauler" or long haul*) adj (COVID or COVID-19 or COVID19 or coronavirus* or corona virus* or 2019-nCoV or 19nCoV or 2019nCoV or nCoV or n-CoV or "CoV 2" or CoV2 or SARS-CoV-2 or SARS-CoV2 or SARSCoV-2 or SARSCoV2 or SARS2 or SARS-2 or severe acute respiratory syndrome coronavirus 2 or 2019-novel CoV or Sars-coronavirus2 or Sars-coronavirus-2 or SARS-like coronavirus* or novel

coronavirus* or novel corona virus* or novel CoV or OC43 or NL63 or 229E or HKU1 or HCoV* or Sars-coronavirus*)).tw,kw,kf. (6323)

16 ((after* or following or post* or "post acute") adj (COVID or COVID-19 or COVID19 or coronavirus* or corona virus* or 2019-nCoV or 19nCoV or 2019nCoV or nCoV or n-CoV or "CoV 2" or CoV2 or SARS-CoV-2 or SARS-CoV2 or SARSCoV-2 or SARSCoV2 or SARS2 or SARS-2 or severe acute respiratory syndrome coronavirus 2 or 2019-novel CoV or Sars-coronavirus2 or Sars-coronavirus-2 or SARS-like coronavirus* or novel coronavirus* or novel corona virus* or novel CoV or OC43 or NL63 or 229E or HKU1 or HCoV* or Sars-coronavirus*) adj3 (chronic* or clinical feature? or comorbid* or "co-morbid*" or complication* or condition* or consequence* or convalescen* or discharge* or disease* or disorder* or effect? or fatigue or followup* or follow-up* or function* or illness* or impact* or infection* or issue* or multimorbid* or "multi morbid*" or nonrecover* or non-recover* or persist* or prognos* or prolonged or recover* or recuperat* or recurr* or rehabilitat* or relaps* or residual or risk* or secondary or sequela* or sickness* or sign or signs or survivor* or survival* or syndrome* or symptom*)).tw,kw,kf. (11464)

17 or/13-16 [PCC -Long Covid Set1] (19807)

18 exp coronavirus disease 2019/ (378565)

19 exp severe acute respiratory syndrome coronavirus 2/ (103741)

20 SARS-CoV-2 variants.mp. (5278)

21 (COVID-19 or COVID19).tw,kw,kf. (399950)

22 (((coronavirus* or corona virus*) and (hubei or wuhan or beijing or shanghai)) or (wuhan adj5 virus*) or (2019-nCoV or 19nCoV or 2019nCoV) or (nCoV or n-CoV or "CoV 2" or CoV2)).tw,kw,kf. (155501)

23 (SARS-CoV-2 or SARS-CoV2 or SARSCoV-2 or SARSCoV2 or SARS2 or SARS-2 or severe acute respiratory syndrome coronavirus 2).tw,kw,kf. (154980)

24 (2019-novel CoV or Sars-coronavirus2 or Sars-coronavirus-2 or SARS-like coronavirus* or ((novel or new or nouveau) adj2 (CoV or nCoV or covid or coronavirus* or corona virus or Pandemi*2)) or (coronavirus* and pneumonia)).tw,kw,kf. (34481)

25 (novel coronavirus* or novel corona virus* or novel CoV).tw,kw,kf. (14783)

26 (((coronavirus* or corona virus*) adj2 "2019") or ((coronavirus* or corona virus*) adj2 "19") or (coronavirus 2 or corona virus 2)).tw,kw,kf. (97304)

27 (OC43 or NL63 or 229E or HKU1 or HCoV* or Sars-coronavirus*).tw,kw,kf. (5458)

28 [COVID-19.rx,px,ox. or severe acute respiratory syndrome coronavirus 2.os.] (0)

29 (coronavirus* or corona virus* or COVID).ti. (317686)

30 or/18-29 [COVID-19] (490808)

31 ((long* or persist* or prolonged or residual) adj8 ((olfactory or chemosensor* or chemo sensor*) adj (disorder* or dysfunction*))).tw,kw,kf. (267)

32 (((post acute or postacute or sub-acute or subacute or chronic) adj sequela*) or PASC or PACS).tw,kw,kf. (10877)
33 (post* adj3 (care or aftercare* or rehabilitati*) adj2 (clinic or clinics or centre or center or centres or centers or program*)).tw,kw,kf. (1962)
34 ((post-intensive care or postintensive care or post-ICU) adj syndrome*).tw,kw,kf. (1096)
35 (recovery adj2 (clinic or clinics or centre or center or centres or centers or program*)).tw,kw,kf. (4045)
36 ((chronic* or continuous* or continual* or continuing* or delay* or endur* or extend* or f
luctuat* or gradual* or lasting* or legacy* or lengthy* or linger* or long* or "medium* term*" or mediumterm* or multisystem* or "multi system*" or ongoing* or permanent* or persist* or prolong* or protract* or relaps* or remission* or remit* or residual* or slow* or subacute* or "sub acute*") adj3 recover*).tw,kw,kf. (47442)
37 ((after discharg* or following discharg* or postacute* or "post acute*" or "post-acute" or postdischarg* or "post discharge" or "post discharging" or posthospital* or post-hospital* or postinfect* or "post infection" or "post infective*" or postviral* or "post viral*" or postvirus* or "post virus*" or postcritical or post-critical or postintensive or post-intensive or post-ICU) adj3 recover*).tw,kw,kf. (1096)
38 ((chronic* or continuous* or continual* or continuing* or delay* or endur* or extend* or f
luctuat* or gradual* or lasting* or legacy* or lengthy* or linger* or long* or "medium* term*" or mediumterm* or multisystem* or "multi system*" or ongoing or permanent* or persist* or prolong* or protract* or relaps* or remission* or remit* or residual* or slow* or subacute* or "sub acute*") adj3 (aftercare* or after-care* or complication? or condition* or consequence? or convalescen* or disabilit* or disease* or disorder* or feature* or illness* or issue* or prognos* or sequela* or sign or signs or suffering? or symptom* or syndrome* or recuperat*)).tw,kw,kf. (1275214)
39 ((after discharg* or following discharg* or late-onset or postacute* or "post acute*" or postdischarg* or "post discharge" or "post discharging" or posthospital* or post-hospital* or postinfect* or "post infection" or "post infective*" or postviral* or "post viral*" or postvirus* or "post virus*" or postcritical or post-critical or postintensive or post-intensive or post-ICU or survivor* or survived) adj3 (complication? or condition* or consequence? or convalescen* or disabilit* or disease* or disorder* or feature* or illness* or issue* or prognos* or sequela* or sign or signs or suffering? or symptom* or syndrome* or recuperat*)).tw,kw,kf. (36083)
40 (nonrecover* or "non recover*" or "not recover*" or ("long* haul*" or long haul* or longhaul* or "long* tail*" or longtail* or longduration* or "long duration*" or longlast* or "long last*" or longstanding* or "long standing*" or "medium* term*" or mediumterm*)).tw,kw,kf.

(161448)
41 or/31-40 [Long haul terms] (1495390)
42 30 and 41 [COVID-19 AND Long Haul: Part 2 of PCC Strategy] (41127)
43 Long Term Adverse.ti,ab,kw,kf. and 30 (140)
44 exp animals/ not exp humans/ (3613357)
45 (2019* or 202*).yr. (9243056)
46 ((or/17,42-43) and 45) not 44 [PCC/Long Covid Sensitive-EM] (51332)
47 12 and 46 (1569)
48 limit 47 to english language (1534)
49 limit 48 to embase (816)
I LOVE EVIDENCE [COVID Segment]
Database: Epistemonikos COVID-19 LOVE Platform
Search Date: 27 February 2024
Filter: Post-COVID-19 conditions
AND
("patient reported outcome*" OR "functional status" OR EuroQol-5D* OR EuroQol5d* OR eq 5d* OR eq5d* OR "fatigue severity" OR "facit-f" OR "long covid symptom and impact" OR PAC 19QoL* OR PAC19QoL* OR PAC-19-QoL* OR Short-Form-36* OR short-form36* OR shortform 36* OR shortform36* OR sf-36* OR sf36*) AND (assess* or instrument* or measur* or questionnaire* or scale* or score* or scoring or test* or tool* or validat*)

<u>Search Strategy Batch #2:</u>

1) In people with suspected PCC, should we use D-dimers versus not use D-dimers?

Ovid MEDLINE(R) ALL <1946 to February 23, 2024>
Search date: Feb-24-2024
1 Fibrin Fibrinogen Degradation Products/ (10161)
2 ((fibrin adj2 degrad* product?) or split product? or (Antithrombin VI or Antithrombin 6)).ti,ab,kw,kf. (3911)
3 d-dimer*.ti,ab,kw,kf. (18211)
4 ddimer*.ti,ab,kw,kf. (55)
5 or/1-4 [D-Dimer] (24074)
6 Post-Acute COVID-19 Syndrome/ (3057)
7 ((chronic* or late sequela* or prolong*) adj2 (COVID or COVID-19 or COVID19 or coronavirus* or corona virus* or 2019-nCoV or 19nCoV or 2019nCoV or nCoV or n-CoV or "CoV 2" or CoV2 or SARS-CoV-2 or SARS-CoV2 or SARSCoV-2 or SARSCoV2 or SARS2 or SARS-2 or severe acute

respiratory syndrome coronavirus 2 or 2019-novel CoV or Sars-coronavirus2 or Sars-coronavirus-2 or SARS-like coronavirus* or novel coronavirus* or novel corona virus* or novel CoV or OC43 or NL63 or 229E or HKU1 or HCoV* or Sars-coronavirus*)).tw,kw,kf. (2734)
8 ((long or longterm* or long-term* or "long haul" or "long haulers" or "long hauler" or long-haul*) adj (COVID or COVID-19 or COVID19 or coronavirus* or corona virus* or 2019-nCoV or 19nCoV or 2019nCoV or nCoV or n-CoV or "CoV 2" or CoV2 or SARS-CoV-2 or SARS-CoV2 or SARSCoV-2 or SARSCoV2 or SARS2 or SARS-2 or severe acute respiratory syndrome coronavirus 2 or 2019-novel CoV or Sars-coronavirus2 or Sars-coronavirus-2 or SARS-like coronavirus* or novel coronavirus* or novel corona virus* or novel CoV or OC43 or NL63 or 229E or HKU1 or HCoV* or Sars-coronavirus*)).tw,kw,kf. (5654)
9 ((after* or following or post* or "post acute") adj (COVID or COVID-19 or COVID19 or coronavirus* or corona virus* or 2019-nCoV or 19nCoV or 2019nCoV or nCoV or n-CoV or "CoV 2" or CoV2 or SARS-CoV-2 or SARS-CoV2 or SARSCoV-2 or SARSCoV2 or SARS2 or SARS-2 or severe acute respiratory syndrome coronavirus 2 or 2019-novel CoV or Sars-coronavirus2 or Sars-coronavirus-2 or SARS-like coronavirus* or novel coronavirus* or novel corona virus* or novel CoV or OC43 or NL63 or 229E or HKU1 or HCoV* or Sars-coronavirus*) adj3 (chronic* or clinical feature? or comorbid* or "co-morbid*" or complication* or condition* or consequence* or convalescen* or discharge* or disease* or disorder* or effect? or fatigue or followup* or follow-up* or function* or illness* or impact* or infection* or issue* or multimorbid* or "multi morbid*" or nonrecover* or non-recover* or persist* or prognos* or prolonged or recover* or recuperat* or recurr* or rehabilitat* or relaps* or residual or risk* or secondary or sequela* or sickness* or sign or signs or survivor* or survival* or syndrome* or symptom*)).tw,kw,kf. (8513)
10 or/6-9 [PCC -Long Covid Set1] (15234)
11 exp Covid-19/ (256062)
12 SARS-CoV-2/ (165671)
Annotation: If multifile: exp COVID-19/ use medall
13 SARS-CoV-2 variants.mp. (10697)
14 (COVID-19 or COVID19).tw,kw,kf. (361928)
15 (((coronavirus* or corona virus*) and (hubei or wuhan or beijing or shanghai)) or (wuhan adj5 virus*) or (2019-nCoV or 19nCoV or 2019nCoV) or (nCoV or n-CoV or "CoV 2" or CoV2)).tw,kw,kf. (141773)
16 (SARS-CoV-2 or SARS-CoV2 or SARSCoV-2 or SARSCoV2 or SARS2 or SARS-2 or severe acute respiratory syndrome coronavirus 2).tw,kw,kf. (141116)
17 (2019-novel CoV or Sars-coronavirus2 or Sars-coronavirus-2 or SARS-like coronavirus* or ((novel or new or nouveau) adj2 (CoV or nCoV or covid or coronavirus* or corona virus or Pandemi*2)) or (coronavirus* and pneumonia)).tw,kw,kf. (31216)
18 (novel coronavirus* or novel corona virus* or novel CoV).tw,kw,kf. (14066)
19 (((coronavirus* or corona virus*) adj2 "2019") or ((coronavirus* or corona virus*) adj2 "19") or (coronavirus 2 or corona virus 2)).tw,kw,kf. (96838)
20 (OC43 or NL63 or 229E or HKU1 or HCoV* or Sars-coronavirus*).tw,kw,kf. (5154)
21 COVID-19.rx,px,ox. or severe acute respiratory syndrome coronavirus 2.os. (2297)
22 (coronavirus* or corona virus* or COVID).ti. (292178)
23 or/11-22 [COVID-19] (419318)
24 ((long* or persist* or prolonged or residual) adj8 ((olfactory or chemosensor* or chemo-sensor*) adj (disorder* or dysfunction*))).tw,kw,kf. (238)
25 (((post acute or postacute or sub-acute or subacute or chronic) adj sequela*) or PASC or PACS).tw,kw,kf. (7797)
26 (post* adj3 (care or aftercare* or rehabilitati*) adj2 (clinic or clinics or centre or center or centres or centers or program*)).tw,kw,kf. (1416)
27 ((post-intensive care or postintensive care or post-ICU) adj syndrome*).tw,kw,kf. (757)

28 (recovery adj2 (clinic or clinics or centre or center or centres or centers or program*)).tw,kw,kf. (2565)
29 ((chronic* or continuous* or continual* or continuing* or delay* or endur* or extend* or fluctuat* or gradual* or lasting* or legacy* or lengthy* or linger* or long* or "medium* term*" or mediumterm* or multisystem* or "multi system*" or ongoing* or permanent* or persist* or prolong* or protract* or relaps* or remission* or remit* or residual* or slow* or subacute* or "sub acute*") adj3 recover*).tw,kw,kf. (39905)
30 ((after discharg* or following discharg* or postacute* or "post acute*" or "post-acute" or postdischarg* or "post discharge" or "post discharging" or posthospital* or post-hospital* or postinfect* or "post infection" or "post infective*" or postviral* or "post viral*" or postvirus* or "post virus*" or postcritical or post-critical or postintensive or post-intensive or post-ICU) adj3 recover*).tw,kw,kf. (781)
31 ((chronic* or continuous* or continual* or continuing* or delay* or endur* or extend* or fluctuat* or gradual* or lasting* or legacy* or lengthy* or linger* or long* or "medium* term*" or mediumterm* or multisystem* or "multi system*" or ongoing or permanent* or persist* or prolong* or protract* or relaps* or remission* or remit* or residual* or slow* or subacute* or "sub acute*") adj3 (aftercare* or after-care* or complication? or condition* or consequence? or convalescen* or disabilit* or disease* or disorder* or feature* or illness* or issue* or prognos* or sequela* or sign or signs or suffering? or symptom* or syndrome* or recuperat*)).tw,kw,kf. (930540)
32 ((after discharg* or following discharg* or late-onset or postacute* or "post acute*" or postdischarg* or "post discharge" or "post discharging" or posthospital* or post-hospital* or postinfect* or "post infection" or "post infective*" or postviral* or "post viral*" or postvirus* or "post virus*" or postcritical or post-critical or postintensive or post-intensive or post-ICU or survivor* or survived) adj3 (complication? or condition* or consequence? or convalescen* or disabilit* or disease* or disorder* or feature* or illness* or issue* or prognos* or sequela* or sign or signs or suffering? or symptom* or syndrome* or recuperat*)).tw,kw,kf. (27845)
33 (nonrecover* or "non recover*" or "not recover*" or ("long* haul*" or long haul* or longhaul* or "long* tail*" or longtail* or longduration* or "long duration*" or longlast* or "long last*" or longstanding* or "long standing*" or "medium* term*" or mediumterm*)).tw,kw,kf. (144486)
34 or/24-33 [Long haul terms] (1123599)
35 23 and 34 [COVID-19 AND Long Haul: Part 2 of PCC Strategy] (30664)
36 Long Term Adverse Effects/ and 23 [Part 3 of PCC sensitive strategy] (11)
37 exp animals/ not exp humans/ (5198554)
38 (2019* or 202*).yr. (7690083)
39 ((or/10,35-36) and 38) not 37 [PCC/Long Covid Sensitive-ML] (38258)
40 5 and 39 (748)
41 limit 40 to english language (730)

EMBASE
Embase <1996 to 2024 February 23>
Search date: Feb-24-2024
1 D dimer/ (43103)
2 d-dimer*.ti,ab,kw,kf. (29992)
3 ddimer*.ti,ab,kw,kf. (925)
4 ((fibrin adj2 degrad* product?) or split product? or (Antithrombin VI or Antithrombin 6)).ti,ab,kw,kf. (2401)
5 or/1-4 [D-Dimer EMBASE] (49091)
6 long COVID/ [EMTREE] (6537)

7 ((chronic* or late sequela* or prolong*) adj2 (COVID or COVID-19 or COVID19 or coronavirus* or corona virus* or 2019-nCoV or 19nCoV or 2019nCoV or nCoV or n-CoV or "CoV 2" or CoV2 or SARS-CoV-2 or SARS-CoV2 or SARSCoV-2 or SARSCoV2 or SARS2 or SARS-2 or severe acute respiratory syndrome coronavirus 2 or 2019-novel CoV or Sars-coronavirus2 or Sars-coronavirus-2 or SARS-like coronavirus* or novel coronavirus* or novel corona virus* or novel CoV or OC43 or NL63 or 229E or HKU1 or HCoV* or Sars-coronavirus*)).tw,kw,kf. (2955)

8 ((long or longterm* or long-term* or "long haul" or "long haulers" or "long hauler" or long-haul*) adj (COVID or COVID-19 or COVID19 or coronavirus* or corona virus* or 2019-nCoV or 19nCoV or 2019nCoV or nCoV or n-CoV or "CoV 2" or CoV2 or SARS-CoV-2 or SARS-CoV2 or SARSCoV-2 or SARSCoV2 or SARS2 or SARS-2 or severe acute respiratory syndrome coronavirus 2 or 2019-novel CoV or Sars-coronavirus2 or Sars-coronavirus-2 or SARS-like coronavirus* or novel coronavirus* or novel corona virus* or novel CoV or OC43 or NL63 or 229E or HKU1 or HCoV* or Sars-coronavirus*)).tw,kw,kf. (6292)

9 ((after* or following or post* or "post acute") adj (COVID or COVID-19 or COVID19 or coronavirus* or corona virus* or 2019-nCoV or 19nCoV or 2019nCoV or nCoV or n-CoV or "CoV 2" or CoV2 or SARS-CoV-2 or SARS-CoV2 or SARSCoV-2 or SARSCoV2 or SARS2 or SARS-2 or severe acute respiratory syndrome coronavirus 2 or 2019-novel CoV or Sars-coronavirus2 or Sars-coronavirus-2 or SARS-like coronavirus* or novel coronavirus* or novel corona virus* or novel CoV or OC43 or NL63 or 229E or HKU1 or HCoV* or Sars-coronavirus*) adj3 (chronic* or clinical feature? or comorbid* or "co-morbid*" or complication* or condition* or consequence* or convalescen* or discharge* or disease* or disorder* or effect? or fatigue or followup* or follow-up* or function* or illness* or impact* or infection* or issue* or multimorbid* or "multi morbid*" or nonrecover* or non-recover* or persist* or prognos* or prolonged or recover* or recuperat* or recurr* or rehabilitat* or relaps* or residual or risk* or secondary or sequela* or sickness* or sign or signs or survivor* or survival* or syndrome* or symptom*)).tw,kw,kf. (11428)

10 or/6-9 [PCC -Long Covid Set1] (19743)

11 exp coronavirus disease 2019/ [EMTREE] (378004)

12 exp severe acute respiratory syndrome coronavirus 2/ [EMTREE] (103558)

13 SARS-CoV-2 variants.mp. (5261)

14 (COVID-19 or COVID19).tw,kw,kf. (399423)

15 (((coronavirus* or corona virus*) and (hubei or wuhan or beijing or shanghai)) or (wuhan adj5 virus*) or (2019-nCoV or 19nCoV or 2019nCoV) or (nCoV or n-CoV or "CoV 2" or CoV2)).tw,kw,kf. (155271)

16 (SARS-CoV-2 or SARS-CoV2 or SARSCoV-2 or SARSCoV2 or SARS2 or SARS-2 or severe acute respiratory syndrome coronavirus 2).tw,kw,kf. (154755)

17 (2019-novel CoV or Sars-coronavirus2 or Sars-coronavirus-2 or SARS-like coronavirus* or ((novel or new or nouveau) adj2 (CoV or nCoV or covid or coronavirus* or corona virus or Pandemi*2)) or (coronavirus* and pneumonia)).tw,kw,kf. (34461)

18 (novel coronavirus* or novel corona virus* or novel CoV).tw,kw,kf. (14775)

19 (((coronavirus* or corona virus*) adj2 "2019") or ((coronavirus* or corona virus*) adj2 "19") or (coronavirus 2 or corona virus 2)).tw,kw,kf. (97201)

20 (OC43 or NL63 or 229E or HKU1 or HCoV* or Sars-coronavirus*).tw,kw,kf. (5454)

21 [COVID-19.rx,px,ox. or severe acute respiratory syndrome coronavirus 2.os.] (0)

22 (coronavirus* or corona virus* or COVID).ti. (317326)

23 or/11-22 [COVID-19] (490184)

24 ((long* or persist* or prolonged or residual) adj8 ((olfactory or chemosensor* or chemo-sensor*) adj (disorder* or dysfunction*))).tw,kw,kf. (266)

25 (((post acute or postacute or sub-acute or subacute or chronic) adj sequela*) or PASC or PACS).tw,kw,kf. (10867)

26 (post* adj3 (care or aftercare* or rehabilitati*) adj2 (clinic or clinics or centre or center or centres or centers or program*)).tw,kw,kf. (1960)

27 ((post-intensive care or postintensive care or post-ICU) adj syndrome*).tw,kw,kf. (1095)
28 (recovery adj2 (clinic or clinics or centre or center or centres or centers or program*)).tw,kw,kf. (4042)
29 ((chronic* or continuous* or continual* or continuing* or delay* or endur* or extend* or fluctuat* or gradual* or lasting* or legacy* or lengthy* or linger* or long* or "medium* term*" or mediumterm* or multisystem* or "multi system*" or ongoing* or permanent* or persist* or prolong* or protract* or relaps* or remission* or remit* or residual* or slow* or subacute* or "sub acute*") adj3 recover*).tw,kw,kf. (47424)
30 ((after discharg* or following discharg* or postacute* or "post acute*" or "post-acute" or postdischarg* or "post discharge" or "post discharging" or posthospital* or post-hospital* or postinfect* or "post infection" or "post infective*" or postviral* or "post viral*" or postvirus* or "post virus*" or postcritical or post-critical or postintensive or post-intensive or post-ICU) adj3 recover*).tw,kw,kf. (1094)
31 ((chronic* or continuous* or continual* or continuing* or delay* or endur* or extend* or fluctuat* or gradual* or lasting* or legacy* or lengthy* or linger* or long* or "medium* term*" or mediumterm* or multisystem* or "multi system*" or ongoing or permanent* or persist* or prolong* or protract* or relaps* or remission* or remit* or residual* or slow* or subacute* or "sub acute*") adj3 (aftercare* or after-care* or complication? or condition* or consequence? or convalescen* or disabilit* or disease* or disorder* or feature* or illness* or issue* or prognos* or sequela* or sign or signs or suffering? or symptom* or syndrome* or recuperat*)).tw,kw,kf. (1274449)
32 ((after discharg* or following discharg* or late-onset or postacute* or "post acute*" or postdischarg* or "post discharge" or "post discharging" or posthospital* or post-hospital* or postinfect* or "post infection" or "post infective*" or postviral* or "post viral*" or postvirus* or "post virus*" or postcritical or post-critical or postintensive or post-intensive or post-ICU or survivor* or survived) adj3 (complication? or condition* or consequence? or convalescen* or disabilit* or disease* or disorder* or feature* or illness* or issue* or prognos* or sequela* or sign or signs or suffering? or symptom* or syndrome* or recuperat*)).tw,kw,kf. (36061)
33 (nonrecover* or "non recover*" or "not recover*" or ("long* haul*" or long haul* or longhaul* or "long* tail*" or longtail* or longduration* or "long duration*" or longlast* or "long last*" or longstanding* or "long standing*" or "medium* term*" or mediumterm*)).tw,kw,kf. (161381)
34 or/24-33 [Long haul terms] (1494537)
35 23 and 34 [COVID-19 AND Long Haul: Part 2 of PCC Strategy] (41054)
36 Long Term Adverse.ti,ab,kw,kf. and 23 [changed to KW since there is no such EMTREE term LTAE] (140)
37 exp animals/ not exp humans/ (3612362)
38 (2019* or 202*).yr. (9228503)
39 ((or/10,35-36) and 38) not 37 [PCC/Long Covid Sensitive-EM] (51223)
40 5 and 39 [D-dimer and PCC sensitive Embase] (2681)
41 limit 40 to english language (2632)

Search Strategy Batch#3:

1) **In people with suspected PCC who have respiratory or cardiac complaints, should we use ambulatory oximetry versus not use ambulatory oximetry?**
2) **In people with suspected PCC who have respiratory or cardiac complaints, should we use an electrocardiogram (ECG) versus not use an ECG?**
3) **In people with suspected PCC who have respiratory or cardiac complaints, should we use a Holter monitor versus not use a Holter monitor**

Database: Ovid MEDLINE(R) ALL <1946 to February 19, 2024>
Search Date: 20 February 2024

----

1 exp oximetry/ (16867)

2 (oximet* or oxymet*).tw,kf,kw. (19576)

3 ((blood gas* or oxygen*) adj2 (monitor* or measur*) adj2 (cutaneous* or transcutan* or noninvas* or non-invas* or skin)).tw,kf,kw. (974)

4 ((transcutan* adj2 (capnometr* or carbon dioxide)) or PTC02 or tcPCO2).tw,kf,kw. (759)

5 or/1-4 [oximetry] (27753)

6 electrocardiography/ (203325)

7 (electrocardiogra* or electro cardiogra*).tw,kf,kw. (112202)

8 (ecg or ecgs or ekg or ekgs).tw,kf,kw. (81822)

9 or/6-8 [ECG] (272269)

10 electrocardiography, ambulatory/ (11635)

11 ((electrocardiogra* or electro cardiogra*) adj3 (ambulatory or dynamic or monitor*)).tw,kf,kw. (7063)

12 holter.tw,kf,kw. (11948)

13 (holter* adj4 (ecg or ecgs or ekg or ekgs or electrocardiograph* or method or monitor* or record*)).tw,kf,kw. (10609)

14 or/10-13 [holter] (23399)

15 5 or 9 or 14 (305303)

16 Post-Acute COVID-19 Syndrome/ (3055)

17 ((chronic* or late sequela* or prolong*) adj2 (COVID or COVID-19 or COVID19 or coronavirus* or corona virus* or 2019-nCoV or 19nCoV or 2019nCoV or nCoV or n-CoV or "CoV 2" or CoV2 or SARS-CoV-2 or SARS-CoV2 or SARSCoV-2 or SARSCoV2 or SARS2 or SARS-2 or severe acute respiratory syndrome coronavirus 2 or 2019-novel CoV or Sars-coronavirus2 or Sars-coronavirus-2 or SARS-like coronavirus* or novel coronavirus* or novel corona virus* or novel CoV or OC43 or NL63 or 229E or HKU1 or HCoV* or Sars-coronavirus*)).tw,kw,kf. (2738)

18 ((long or longterm* or long-term* or "long haul" or "long haulers" or "long hauler" or long-haul*) adj (COVID or COVID-19 or COVID19 or coronavirus* or corona virus* or 2019-nCoV or 19nCoV or 2019nCoV or nCoV or n-CoV or "CoV 2" or CoV2 or SARS-CoV-2 or SARS-CoV2 or SARSCoV-2 or SARSCoV2 or SARS2 or SARS-2 or severe acute respiratory syndrome coronavirus 2 or 2019-novel CoV or Sars-coronavirus2 or Sars-coronavirus-2 or SARS-like coronavirus* or novel coronavirus* or novel corona virus* or novel CoV or OC43 or NL63 or 229E or HKU1 or HCoV* or Sars-coronavirus*)).tw,kw,kf. (5650)
19 ((after* or following or post* or "post acute") adj (COVID or COVID-19 or COVID19 or coronavirus* or corona virus* or 2019-nCoV or 19nCoV or 2019nCoV or nCoV or n-CoV or "CoV 2" or CoV2 or SARS-CoV-2 or SARS-CoV2 or SARSCoV-2 or SARSCoV2 or SARS2 or SARS-2 or severe acute respiratory syndrome coronavirus 2 or 2019-novel CoV or Sars-coronavirus2 or Sars-coronavirus-2 or SARS-like coronavirus* or novel coronavirus* or novel corona virus* or novel CoV or OC43 or NL63 or 229E or HKU1 or HCoV* or Sars-coronavirus*) adj3 (chronic* or clinical feature? or comorbid* or "co-morbid*" or complication* or condition* or consequence* or convalescen* or discharge* or disease* or disorder* or effect? or fatigue or followup* or follow-up* or function* or illness* or impact* or infection* or issue* or multimorbid* or "multi morbid*" or nonrecover* or non-recover* or persist* or prognos* or prolonged or recover* or recuperat* or recurr* or rehabilitat* or relaps* or residual or risk* or secondary or sequela* or sickness* or sign or signs or survivor* or survival* or syndrome* or symptom*)).tw,kw,kf. (8503)
20 or/16-19 [PCC -Long Covid Set1] (15219)
21 exp Covid-19/ (256269)
22 SARS-CoV-2/ (165825)
23 SARS-CoV-2 variants.mp. (10748)
24 (COVID-19 or COVID19).tw,kw,kf. (362066)
25 (((coronavirus* or corona virus*) and (hubei or wuhan or beijing or shanghai)) or (wuhan adj5 virus*) or (2019-nCoV or 19nCoV or 2019nCoV) or (nCoV or n-CoV or "CoV 2" or CoV2)).tw,kw,kf. (141936)
26 (SARS-CoV-2 or SARS-CoV2 or SARSCoV-2 or SARSCoV2 or SARS2 or SARS-2 or severe acute respiratory syndrome coronavirus 2).tw,kw,kf. (141285)
27 (2019-novel CoV or Sars-coronavirus2 or Sars-coronavirus-2 or SARS-like coronavirus* or ((novel or new or nouveau) adj2 (CoV or nCoV or covid or coronavirus* or corona virus or Pandemi*2)) or (coronavirus* and pneumonia)).tw,kw,kf. (31190)
28 (novel coronavirus* or novel corona virus* or novel CoV).tw,kw,kf. (14062)
29 (((coronavirus* or corona virus*) adj2 "2019") or ((coronavirus* or corona virus*) adj2 "19") or (coronavirus 2 or corona virus 2)).tw,kw,kf. (96850)
30 (OC43 or NL63 or 229E or HKU1 or HCoV* or Sars-coronavirus*).tw,kw,kf. (5161)
31 COVID-19.rx,px,ox. or severe acute respiratory syndrome coronavirus 2.os. (2297)
32 (coronavirus* or corona virus* or COVID).ti. (292250)
33 or/21-32 [COVID-19] (419528)
34 ((long* or persist* or prolonged or residual) adj8 ((olfactory or chemosensor* or chemo-sensor*) adj (disorder* or dysfunction*))).tw,kw,kf. (239)
35 (((post acute or postacute or sub-acute or subacute or chronic) adj sequela*) or PASC or PACS).tw,kw,kf. (7814)
36 (post* adj3 (care or aftercare* or rehabilitati*) adj2 (clinic or clinics or centre or center or centres or centers or program*)).tw,kw,kf. (1416)
37 ((post-intensive care or postintensive care or post-ICU) adj syndrome*).tw,kw,kf. (754)
38 (recovery adj2 (clinic or clinics or centre or center or centres or centers or program*)).tw,kw,kf. (2571)

39 ((chronic* or continuous* or continual* or continuing* or delay* or endur* or extend* or fluctuat* or gradual* or lasting* or legacy* or lengthy* or linger* or long* or "medium* term*" or mediumterm* or multisystem* or "multi system*" or ongoing* or permanent* or persist* or prolong* or protract* or relaps* or remission* or remit* or residual* or slow* or subacute* or "sub acute*") adj3 recover*).tw,kw,kf. (39890)
40 ((after discharg* or following discharg* or postacute* or "post acute*" or "post-acute" or postdischarg* or "post discharge" or "post discharging" or posthospital* or post-hospital* or postinfect* or "post infection" or "post infective*" or postviral* or "post viral*" or postvirus* or "post virus*" or postcritical or post-critical or postintensive or post-intensive or post-ICU) adj3 recover*).tw,kw,kf. (779)
41 ((chronic* or continuous* or continual* or continuing* or delay* or endur* or extend* or fluctuat* or gradual* or lasting* or legacy* or lengthy* or linger* or long* or "medium* term*" or mediumterm* or multisystem* or "multi system*" or ongoing or permanent* or persist* or prolong* or protract* or relaps* or remission* or remit* or residual* or slow* or subacute* or "sub acute*") adj3 (aftercare* or after-care* or complication? or condition* or consequence? or convalescen* or disabilit* or disease* or disorder* or feature* or illness* or issue* or prognos* or sequela* or sign or signs or suffering? or symptom* or syndrome* or recuperat*)).tw,kw,kf. (930600)
42 ((after discharg* or following discharg* or late-onset or postacute* or "post acute*" or postdischarg* or "post discharge" or "post discharging" or posthospital* or post-hospital* or postinfect* or "post infection" or "post infective*" or postviral* or "post viral*" or postvirus* or "post virus*" or postcritical or post-critical or postintensive or post-intensive or post-ICU or survivor* or survived) adj3 (complication? or condition* or consequence? or convalescen* or disabilit* or disease* or disorder* or feature* or illness* or issue* or prognos* or sequela* or sign or signs or suffering? or symptom* or syndrome* or recuperat*)).tw,kw,kf. (27855)
43 (nonrecover* or "non recover*" or "not recover*" or ("long* haul*" or long haul* or longhaul* or "long* tail*" or longtail* or longduration* or "long duration*" or longlast* or "long last*" or longstanding* or "long standing*" or "medium* term*" or mediumterm*)).tw,kw,kf. (144521)
44 or/34-43 [Long haul terms] (1123690)
45 33 and 44 [COVID-19 AND Long Haul: Part 2 of PCC Strategy] (30694)
46 Long Term Adverse Effects/ and 33 [Part 3 of PCC sensitive strategy] (11)
47 exp animals/ not exp humans/ (5199285)
48 (2019* or 202*).yr. (7689654)
49 ((or/20,45-46) and 48) not 47 [PCC/Long Covid Sensitive-ML] (38272)
50 15 and 49 (455)
51 limit 50 to english language (436)

Database: Embase <1996 to 2024 February 19>
Search Date: 20 February 2024

----

1 exp oximetry/ (31904)
2 (oximet* or oxymet*).tw,kf,kw. (23787)
3 ((blood gas* or oxygen*) adj2 (monitor* or measur*) adj2 (cutaneous* or transcutan* or noninvas* or non-invas* or skin)).tw,kf,kw. (726)
4 ((transcutan* adj2 (capnometr* or carbon dioxide)) or PTC02 or tcPCO2).tw,kf,kw. (858)
5 or/1-4 [oximetry] (40325)

6 exp electrocardiography/ (144989)
7 (electrocardiogra* or electro cardiogra*).tw,kf,kw. (105861)
8 (ecg or ecgs or ekg or ekgs).tw,kf,kw. (128197)
9 or/6-8 [ecg] (262925)
10 ambulatory electrocardiography/ (570)
11 ((electrocardiogra* or electro cardiogra*) adj3 (ambulatory or dynamic or monitor*)).tw,kf,kw. (6399)
12 holter monitor/ or holter monitoring/ (19311)
13 holter.tw,kf,kw. (18018)
14 (holter* adj4 (ecg or ecgs or ekg or ekgs or electrocardiograph* or method or monitor* or record*)).tw,kf,kw. (15803)
15 or/10-14 (30780)
16 5 or 9 or 15 (301201)
17 Long COVID/ (6468)
18 ((chronic* or late sequela* or prolong*) adj2 (COVID or COVID-19 or COVID19 or coronavirus* or corona virus* or 2019-nCoV or 19nCoV or 2019nCoV or nCoV or n-CoV or "CoV 2" or CoV2 or SARS-CoV-2 or SARS-CoV2 or SARSCoV-2 or SARSCoV2 or SARS2 or SARS-2 or severe acute respiratory syndrome coronavirus 2 or 2019-novel CoV or Sars-coronavirus2 or Sars-coronavirus-2 or SARS-like coronavirus* or novel coronavirus* or novel corona virus* or novel CoV or OC43 or NL63 or 229E or HKU1 or HCoV* or Sars-coronavirus*)).ti,kw,kf. (2262)
19 ((long or longterm* or long-term* or "long haul" or "long haulers" or "long hauler" or long-haul*) adj (COVID or COVID-19 or COVID19 or coronavirus* or corona virus* or 2019-nCoV or 19nCoV or 2019nCoV or nCoV or n-CoV or "CoV 2" or CoV2 or SARS-CoV-2 or SARS-CoV2 or SARSCoV-2 or SARSCoV2 or SARS2 or SARS-2 or severe acute respiratory syndrome coronavirus 2 or 2019-novel CoV or Sars-coronavirus2 or Sars-coronavirus-2 or SARS-like coronavirus* or novel coronavirus* or novel corona virus* or novel CoV or OC43 or NL63 or 229E or HKU1 or HCoV* or Sars-coronavirus*)).ti,kw,kf. (4806)
20 ((after* or following or post* or "post acute") adj (COVID or COVID-19 or COVID19 or coronavirus* or corona virus* or 2019-nCoV or 19nCoV or 2019nCoV or nCoV or n-CoV or "CoV 2" or CoV2 or SARS-CoV-2 or SARS-CoV2 or SARSCoV-2 or SARSCoV2 or SARS2 or SARS-2 or severe acute respiratory syndrome coronavirus 2 or 2019-novel CoV or Sars-coronavirus2 or Sars-coronavirus-2 or SARS-like coronavirus* or novel coronavirus* or novel corona virus* or novel CoV or OC43 or NL63 or 229E or HKU1 or HCoV* or Sars-coronavirus*) adj3 (chronic* or clinical feature? or comorbid* or "co-morbid*" or complication* or condition* or consequence* or convalescen* or discharge* or disease* or disorder* or effect? or fatigue or followup* or follow-up* or function* or illness* or impact* or infection* or issue* or multimorbid* or "multi morbid*" or nonrecover* or non-recover* or persist* or prognos* or prolonged or recover* or recuperat* or recurr* or rehabilitat* or relaps* or residual or risk* or secondary or sequela* or sickness* or sign or signs or survivor* or survival* or syndrome* or symptom*)).ti,kw,kf. (5590)
21 or/17-20 [Part 1] (13970)
22 exp *severe acute respiratory syndrome coronavirus 2/ (42099)
23 exp *coronavirus disease 2019/ (297111)
24 SARS-CoV-2 variants.ti,hw,kf,kw. (1986)
25 (COVID-19 or COVID19).ti,kw,kf. (325013)
26 (((coronavirus* or corona virus*) and (hubei or wuhan or beijing or shanghai)) or (wuhan adj5 virus*)).ti,kw,kf. (669)
27 (2019-nCoV or 19nCoV or 2019nCoV or (nCoV or n-CoV or "CoV 2" or CoV2)).ti,kw,kf. (103312)

28 (SARS-CoV-2 or SARS-CoV2 or SARSCoV-2 or SARSCoV2 or SARS2 or SARS-2 or severe acute respiratory syndrome coronavirus 2).ti,kw,kf. (105201)
29 (2019-novel CoV or Sars-coronavirus2 or Sars-coronavirus-2 or SARS-like coronavirus* or ((novel or new or nouveau) adj2 (CoV or nCoV or covid or coronavirus* or corona virus or Pandemi*2)) or (coronavirus* and pneumonia)).ti,kw,kf. (8146)
30 (novel coronavirus* or novel corona virus* or novel CoV or ((coronavirus* or corona virus*) adj2 "2019") or ((coronavirus* or corona virus*) adj2 "19")).ti,kw,kf. (23384)
31 (coronavirus 2 or corona virus 2).ti,kw,kf. (6754)
32 (OC43 or NL63 or 229E or HKU1 or HCoV* or Sars-coronavirus*).ti,kw,kf. (2077)
33 [COVID-19.rx,px,ox. or severe acute respiratory syndrome coronavirus 2.os.] (0)
34 (coronavirus* or corona virus* or COVID).ti. (316477)
35 or/23-34 [Covid 19] (403761)
36 ((long* or persist* or prolonged or residual) adj8 ((olfactory or chemosensor* or chemo-sensor*) adj (disorder* or dysfunction*))).ti,kw,kf. (108)
37 (((post acute or postacute or sub-acute or subacute or chronic) adj sequela*) or PASC or PACS).ti,kw,kf. (2756)
38 (post* adj3 (care or aftercare* or rehabilitati*) adj2 (clinic or clinics or centre or center or centres or centers or program*)).ti,kw,kf. (298)
39 ((post-intensive care or postintensive care or post-ICU) adj syndrome*).ti,kw,kf. (683)
40 (recovery adj2 (clinic or clinics or centre or center or centres or centers or program*)).ti,kw,kf. (1410)
41 ((chronic* or continuous* or continual* or continuing* or delay* or endur* or extend* or fluctuat* or gradual* or lasting* or legacy* or lengthy* or linger* or long* or "medium* term*" or mediumterm* or multisystem* or "multi system*" or ongoing* or permanent* or persist* or prolong* or protract* or relaps* or remission* or remit* or residual* or slow* or subacute* or "sub acute*") adj3 recover*).ti,kw,kf. (4893)
42 ((after discharg* or following discharg* or postacute* or "post acute*" or "post-acute" or postdischarg* or "post discharge" or "post discharging" or posthospital* or post-hospital* or postinfect* or "post infection" or "post infective*" or postviral* or "post viral*" or postvirus* or "post virus*" or postcritical or post-critical or postintensive or post-intensive or post-ICU) adj3 recover*).ti,kw,kf. (163)
43 ((chronic* or continuous* or continual* or continuing* or delay* or endur* or extend* or fluctuat* or gradual* or lasting* or legacy* or lengthy* or linger* or long* or "medium* term*" or mediumterm* or multisystem* or "multi system*" or ongoing or permanent* or persist* or prolong* or protract* or relaps* or remission* or remit* or residual* or slow* or subacute* or "sub acute*") adj3 (aftercare* or after-care* or complication? or condition* or consequence? or convalescen* or disabilit* or disease* or disorder* or feature* or illness* or issue* or prognos* or sequela* or sign or signs or suffering? or symptom* or syndrome* or recuperat*)).ti,kw,kf. (267335)
44 ((after discharg* or following discharg* or late-onset or postacute* or "post acute*" or postdischarg* or "post discharge" or "post discharging" or posthospital* or post-hospital* or postinfect* or "post infection" or "post infective*" or postviral* or "post viral*" or postvirus* or "post virus*" or postcritical or post-critical or postintensive or post-intensive or post-ICU or survivor* or survived) adj3 (complication? or condition* or consequence? or convalescen* or disabilit* or disease* or disorder* or feature* or illness* or issue* or prognos* or sequela* or sign or signs or suffering? or symptom* or syndrome* or recuperat*)).ti,kw,kf. (9298)
45 (nonrecover* or "non recover*" or "not recover*").ti,kw,kf. (198)
46 ("long* haul*" or long haul* or longhaul* or "long* tail*" or longtail* or longduration* or "long duration*" or longlast* or "long last*" or longstanding* or "long standing*" or "medium* term*" or mediumterm*).ti,kw,kf. (16179)
47 or/36-46 [Long term AE] (299549)
48 and/35,47 [Part 2] (9382)

49 21 or 48 [PCC/Long Covid Precise Medline] (19373)
50 exp animals/ not exp humans/ (3608882)
51 (2019* or 202*).yr. (9182964)
52 (and/49,51) not 50 [PCC Long Covid Precise ML] (19296)
53 16 and 52 (623)
54 limit 53 to english language (607)
55 limit 54 to embase (406)

Database: Cochrane COVID-19 Study Register
Search Date: 20 February 2024
Epidemiology (prevalence and incidence) filter
AND
(ecg* OR ekg* OR electrocardiog* OR holter* OR oximet* OR oxymet* OR PTC02 OR tcPCO2) (282)
OR
Post-COVID-19 conditions filter
AND
(ecg* OR ekg* OR electrocardiog* OR holter* OR oximet* OR oxymet* OR PTC02 OR tcPCO2) (121)

Database: Epistemonikos COVID-19 LOVE Platform
Search Date: 20 February 2024

Epidemiology filter
AND
(ecg* OR ekg* OR electrocardiog* OR holter* OR oximet* OR oxymet* OR PTC02 OR tcPCO2) (174)

<u>Search Strategy Batch#4:</u>

1)In people with suspected PCC, should we use C-reactive protein (CRP), CRP test versus not use CRP?

2) In people with suspected PCC who have respiratory or cardiac complaints, should we use a chest x-ray versus not use a chest x-ray?

3) In people with suspected PCC who have respiratory or cardiac complaints, should we use an echocardiogram versus not use an echocardiogram?

4) In people with suspected PCC who have respiratory or cardiac complaints, should we use spirometry and full lung function tests (PFTs), DLCO versus not use spirometry and full lung function tests?

5) In people with suspected PCC who have respiratory or cardiac complaints, should we use computed tomography of the chest (CT Chest) versus not use CT of the chest?

6) In people with suspected PCC who have respiratory or cardiac complaints, should we use B-type natriuretic peptide (BNP)/NT-proBNP, BNP test, B-type natriuretic peptide test versus not use BNP/NT-proBNP?

7) In people with suspected PCC who have respiratory or cardiac complaints, should we use Troponin (High sensitive Troponin I) versus not use Troponin?

8) In people with suspected PCC, should we use iron studies (iron saturation and ferritin- higher threshold) versus not use iron studies?

9) In people with suspected PCC who have dizziness, should we use the 6 or 10-minute standing tests versus not using these tests?

10) In people with suspected PCC who have fatigue, should we use DePaul Post Exertional Malaise Questionnaire (DPEMQ) to assess post exertional malaise versus not use these validated tests to assess post exertional malaise?

Database: Ovid MEDLINE(R) <1996 to March 18, 2024>
Search Date: 19 March 2024

----

1 *c-reactive protein/ (17622)
2 (c-reactive protein or creactive protein).tw,kf,kw. (75686)
3 (crp adj3 (kit? or level? or measur* or test* or value?)).tw,kf,kw. (21243)
4 (crp adj3 (immunoassay or immunodiffusion or nephelometry or spectrophotometry)).tw,kf,kw. (112)
5 serum crp.tw,kf,kw. (3039)
6 or/1-5 [CRP] (81834)
7 exp *radiography, thoracic/ (5924)
8 (thora* adj4 (radiogra* or xray* or x-ray*)).tw,kf,kw. (3161)

9 thora* film*.tw,kf,kw. (3)
10 (chest adj4 (radiogra* or xray* or x-ray*)).tw,kf,kw. (33850)
11 chest film*.tw,kf,kw. (383)
12 CXR.tw,kf,kw. (2555)
13 or/7-12 [chest x-ray] (39616)
14 exp *echocardiography/ (37790)
15 (echocardiog* or echo-cardiog*).tw,kf,kw. (130961)
16 (cardiac adj2 (scan* or ultrasound*)).tw,kf,kw. (2369)
17 or/14-16 [echocardiogram] (139238)
18 vital capacity/ (10611)
19 vital capacit*.tw,kf,kw. (13946)
20 forced expiratory volume/ (21159)
21 ((forced or timed) adj2 (expirat* volume* or vital capacit*)).tw,kf,kw. (21776)
22 *spirometry/ (2212)
23 (spiromet* or bronchospiromet*).tw,kf,kw. (19708)
24 ((lung? or pulmonar*) adj2 (function* or capacit*) adj2 (measur* or test*)).tw,kf,kw. (17122)
25 (diffus* capacit* adj3 carbon monoxide).tw,kf,kw. (1973)
26 (DLCO or TLCO).tw,kf,kw. (3185)
27 or/18-26 [lung function tests] (58681)
28 tomograph*.hw. and (exp thorax/ or exp lung/) (23299)
29 tomograph*.hw. and (cardiac or chest or lung* or pulmonary or thora*).tw,kf,kw. (108032)
30 tomograph*.tw,kf,kw. (381218)
31 ((ct or cat) adj5 (cardiac or chest or lung* or pulmonary or thora*)).tw,kf,kw. (38267)
32 or/28-31 [chest tomography] (446118)
33 natriuretic.hw. and (BNP or ntpprobnp or NT-proBNP or proBNP).tw,kf,kw. (11980)
34 ((BNP or ntpprobnp or NT-proBNP or proBNP) adj3 (level? or measur* or test* or value?)).tw,kf,kw. (8800)
35 or/33-34 [BNP test] (13874)
36 troponin.hw. and (level? or measur* or test* or value?).tw,kf,kw. (12605)
37 high sensitiv* troponin*.tw,kf,kw. (2366)
38 or/36-37 [troponin test] (13487)
39 exp iron/ and hematological tests/ (107)
40 exp iron/ and (level? or measur* or saturation or studies or study or test* or value?).tw,kf,kw. (47236)
41 ferritins.hw. and (level? or measur* or saturation or studies or study or test* or value?).tw,kf,kw. (11297)
42 ((ferritin* or iron?) adj5 (level? or measur* or saturation or studies or study or test* or value?)).tw,kf,kw. (29218)
43 blood test*.tw,kf,kw. (20087)
44 or/39-43 [iron tests] (83794)
45 stand test*.tw,kf,kw. (2250)

46 standing test*.tw,kf,kw. (412)
47 or/45-46 [standing test] (2638)
48 (postexertion* malais* or post-exertion* malais*).tw,kf,kw. (215)
49 DPEMQ.mp. (0)
50 or/48-49 [DePaul test] (215)
51 6 or 13 or 17 or 27 or 32 or 35 or 38 or 44 or 47 or 50 [all tests] (819383)
52 Post-Acute COVID-19 Syndrome/ (3154)
53 ((chronic* or late sequela* or prolong*) adj2 (COVID or COVID-19 or COVID19 or coronavirus* or corona virus* or 2019-nCoV or 19nCoV or 2019nCoV or nCoV or n-CoV or "CoV 2" or CoV2 or SARS-CoV-2 or SARS-CoV2 or SARSCoV-2 or SARSCoV2 or SARS2 or SARS-2 or severe acute respiratory syndrome coronavirus 2 or 2019-novel CoV or Sars-coronavirus2 or Sars-coronavirus-2 or SARS-like coronavirus* or novel coronavirus* or novel corona virus* or novel CoV or OC43 or NL63 or 229E or HKU1 or HCoV* or Sars-coronavirus*)).tw,kw,kf. (1742)
54 ((long or longterm* or long-term* or "long haul" or "long haulers" or "long hauler" or long-haul*) adj (COVID or COVID-19 or COVID19 or coronavirus* or corona virus* or 2019-nCoV or 19nCoV or 2019nCoV or nCoV or n-CoV or "CoV 2" or CoV2 or SARS-CoV-2 or SARS-CoV2 or SARSCoV-2 or SARSCoV2 or SARS2 or SARS-2 or severe acute respiratory syndrome coronavirus 2 or 2019-novel CoV or Sars-coronavirus2 or Sars-coronavirus-2 or SARS-like coronavirus* or novel coronavirus* or novel corona virus* or novel CoV or OC43 or NL63 or 229E or HKU1 or HCoV* or Sars-coronavirus*)).tw,kw,kf. (3719)
55 ((after* or following or post* or "post acute") adj (COVID or COVID-19 or COVID19 or coronavirus* or corona virus* or 2019-nCoV or 19nCoV or 2019nCoV or nCoV or n-CoV or "CoV 2" or CoV2 or SARS-CoV-2 or SARS-CoV2 or SARSCoV-2 or SARSCoV2 or SARS2 or SARS-2 or severe acute respiratory syndrome coronavirus 2 or 2019-novel CoV or Sars-coronavirus2 or Sars-coronavirus-2 or SARS-like coronavirus* or novel coronavirus* or novel corona virus* or novel CoV or OC43 or NL63 or 229E or HKU1 or HCoV* or Sars-coronavirus*) adj3 (chronic* or clinical feature? or comorbid* or "co-morbid*" or complication* or condition* or consequence* or convalescen* or discharge* or disease* or disorder* or effect? or fatigue or followup* or follow-up* or function* or illness* or impact* or infection* or issue* or multimorbid* or "multi morbid*" or nonrecover* or non-recover* or persist* or prognos* or prolonged or recover* or recuperat* or recurr* or rehabilitat* or relaps* or residual or risk* or secondary or sequela* or sickness* or sign or signs or survivor* or survival* or syndrome* or symptom*)).tw,kw,kf. (5302)
56 or/52-55 [PCC -Long Covid Set1] (9818)
57 exp Covid-19/ (257727)
58 SARS-CoV-2/ (166032)
59 SARS-CoV-2 variants.mp. (8934)
60 (COVID-19 or COVID19).tw,kw,kf. (237854)
61 (((coronavirus* or corona virus*) and (hubei or wuhan or beijing or shanghai)) or (wuhan adj5 virus*) or (2019-nCoV or 19nCoV or 2019nCoV) or (nCoV or n-CoV or "CoV 2" or CoV2)).tw,kw,kf. (97300)
62 (SARS-CoV-2 or SARS-CoV2 or SARSCoV-2 or SARSCoV2 or SARS2 or SARS-2 or severe acute respiratory syndrome coronavirus 2).tw,kw,kf. (97026)
63 (2019-novel CoV or Sars-coronavirus2 or Sars-coronavirus-2 or SARS-like coronavirus* or ((novel or new or nouveau) adj2 (CoV or nCoV or covid or coronavirus* or corona virus or Pandemi*2)) or (coronavirus* and pneumonia)).tw,kw,kf. (20428)
64 (novel coronavirus* or novel corona virus* or novel CoV).tw,kw,kf. (9084)
65 (((coronavirus* or corona virus*) adj2 "2019") or ((coronavirus* or corona virus*) adj2 "19") or (coronavirus 2 or corona virus 2)).tw,kw,kf. (64193)

66 (OC43 or NL63 or 229E or HKU1 or HCoV* or Sars-coronavirus*).tw,kw,kf. (4051)
67 COVID-19.rx,px,ox. or severe acute respiratory syndrome coronavirus 2.os. (2325)
68 (coronavirus* or corona virus* or COVID).ti. (193468)
69 or/57-68 [COVID-19] (280985)
70 ((long* or persist* or prolonged or residual) adj8 ((olfactory or chemosensor* or chemo-sensor*) adj (disorder* or dysfunction*))).tw,kw,kf. (173)
71 (((post acute or postacute or sub-acute or subacute or chronic) adj sequela*) or PASC or PACS).tw,kw,kf. (5654)
72 (post* adj3 (care or aftercare* or rehabilitati*) adj2 (clinic or clinics or centre or center or centres or centers or program*)).tw,kw,kf. (1066)
73 ((post-intensive care or postintensive care or post-ICU) adj syndrome*).tw,kw,kf. (550)
74 (recovery adj2 (clinic or clinics or centre or center or centres or centers or program*)).tw,kw,kf. (1987)
75 ((chronic* or continuous* or continual* or continuing* or delay* or endur* or extend* or fluctuat* or gradual* or lasting* or legacy* or lengthy* or linger* or long* or "medium* term*" or mediumterm* or multisystem* or "multi system*" or ongoing* or permanent* or persist* or prolong* or protract* or relaps* or remission* or remit* or residual* or slow* or subacute* or "sub acute*") adj3 recover*).tw,kw,kf. (27655)
76 ((after discharg* or following discharg* or postacute* or "post acute*" or "post-acute" or postdischarg* or "post discharge" or "post discharging" or posthospital* or post-hospital* or postinfect* or "post infection" or "post infective*" or postviral* or "post viral*" or postvirus* or "post virus*" or postcritical or post-critical or postintensive or post-intensive or post-ICU) adj3 recover*).tw,kw,kf. (610)
77 ((chronic* or continuous* or continual* or continuing* or delay* or endur* or extend* or fluctuat* or gradual* or lasting* or legacy* or lengthy* or linger* or long* or "medium* term*" or mediumterm* or multisystem* or "multi system*" or ongoing or permanent* or persist* or prolong* or protract* or relaps* or remission* or remit* or residual* or slow* or subacute* or "sub acute*") adj3 (aftercare* or after-care* or complication? or condition* or consequence? or convalescen* or disabilit* or disease* or disorder* or feature* or illness* or issue* or prognos* or sequela* or sign or signs or suffering? or symptom* or syndrome* or recuperat*)).tw,kw,kf. (684857)
78 ((after discharg* or following discharg* or late-onset or postacute* or "post acute*" or postdischarg* or "post discharge" or "post discharging" or posthospital* or post-hospital* or postinfect* or "post infection" or "post infective*" or postviral* or "post viral*" or postvirus* or "post virus*" or postcritical or post-critical or postintensive or post-intensive or post-ICU or survivor* or survived) adj3 (complication? or condition* or consequence? or convalescen* or disabilit* or disease* or disorder* or feature* or illness* or issue* or prognos* or sequela* or sign or signs or suffering? or symptom* or syndrome* or recuperat*)).tw,kw,kf. (20603)
79 (nonrecover* or "non recover*" or "not recover*" or ("long* haul*" or long haul* or longhaul* or "long* tail*" or longtail* or longduration* or "long duration*" or longlast* or "long last*" or longstanding* or "long standing*" or "medium* term*" or mediumterm*)).tw,kw,kf. (98956)
80 or/70-79 [Long haul terms] (818636)
81 69 and 80 [COVID-19 AND Long Haul: Part 2 of PCC Strategy] (20273)
82 Long Term Adverse Effects/ and 69 [Part 3 of PCC sensitive strategy] (11)
83 exp animals/ not exp humans/ (2988349)
84 (2019* or 202*).yr. (5195770)
85 ((or/56,81-82) and 84) not 83 [PCC/Long Covid Sensitive-ML] (25109)
86 (systematic review or meta-analysis).pt. (330732)
87 meta-analysis/ or systematic review/ or systematic reviews as topic/ or meta-analysis as topic/ or "meta analysis (topic)"/ or "systematic review (topic)"/ or exp technology assessment, biomedical/ or network meta-analysis/ (367473)
88 ((systematic* adj3 (review* or overview*)) or (methodologic* adj3 (review* or overview*))).ti,ab,kf. (279507)

89 ((quantitative adj3 (review* or overview* or synthes*)) or (research adj3 (integrati* or overview*))).ti,ab,kf. (12645)
90 ((integrative adj3 (review* or overview*)) or (collaborative adj3 (review* or overview*)) or (pool* adj3 analy*)).ti,ab,kf. (34343)
91 (data synthes* or data extraction* or data abstraction*).ti,ab,kf. (35879)
92 (handsearch* or hand search*).ti,ab,kf. (10091)
93 (mantel haenszel or peto or der simonian or dersimonian or fixed effect* or latin square*).ti,ab,kf. (30285)
94 (met analy* or metanaly* or technology assessment* or HTA or HTAs or technology overview* or technology appraisal*).ti,ab,kf. (9636)
95 (meta regression* or metaregression*).ti,ab,kf. (13417)
96 (meta-analy* or metaanaly* or systematic review* or biomedical technology assessment* or bio-medical technology assessment*).mp,hw. (413609)
97 (medline or cochrane or pubmed or medlars or embase or cinahl).ti,ab,hw. (298031)
98 (cochrane or (health adj2 technology assessment) or evidence report).jw. (20358)
99 (comparative adj3 (efficacy or effectiveness)).ti,ab,kf. (13856)
100 (outcomes research or relative effectiveness).ti,ab,kf. (8876)
101 ((indirect or indirect treatment or mixed-treatment or bayesian) adj3 comparison*).ti,ab,kf. (3547)
102 [(meta-analysis or systematic review).md.] (0)
103 (multi* adj3 treatment adj3 comparison*).ti,ab,kf. (246)
104 (mixed adj3 treatment adj3 (meta-analy* or metaanaly*)).ti,ab,kf. (160)
105 umbrella review*.ti,ab,kf. (1429)
106 (multi* adj2 paramet* adj2 evidence adj2 synthesis).ti,ab,kf. (12)
107 (multiparamet* adj2 evidence adj2 synthesis).ti,ab,kf. (17)
108 (multi-paramet* adj2 evidence adj2 synthesis).ti,ab,kf. (10)
109 or/86-108 [SR-MA-HTA- CADTH- ML-EM-PI https://searchfilters.cadth.ca/link/33] (586179)
110 randomized controlled trial.pt. (515373)
111 controlled clinical trial.pt. (50276)
112 Clinical Trials as topic.sh. (119914)
113 trial.ti,ab. (614459)
114 factorial*.ti,ab. (28886)
115 (crossover* or cross over*).ti,ab. (68149)
116 ((doubl* or singl*) adj blind*).ti,ab. (135384)
117 (assign* or allocat* or volunteer* or placebo*).ti,ab. (702423)
118 placebo.ab. (181850)
119 random*.ti,ab. (1132390)
120 or/110-119 (1984644)
121 animals/ not humans/ (2985060)
122 exp Animals, Laboratory/ (573106)
123 exp Animal Experimentation/ (8250)
124 exp Models, Animal/ (489561)
125 exp Rodentia/ (2261495)

126 (rat or rats or mouse or mice).ti. (774469)
127 or/121-126 (3757403)
128 120 not 127 [RCT ML] (1752394)
129 epidemiologic methods.sh. (17293)
130 epidemiologic studies.sh. (8827)
131 observational study/ (153290)
132 observational studies as topic/ (9552)
133 clinical studies as topic/ (822)
134 controlled before-after studies/ (752)
135 cross-sectional studies/ (476776)
136 historically controlled study/ (232)
137 interrupted time series analysis/ (2008)
138 exp seroepidemiologic studies/ (25297)
139 national longitudinal study of adolescent health/ (103)
140 cohort studies/ (326368)
141 cohort analysis/ (326368)
142 longitudinal studies/ (153403)
143 longitudinal study/ (153403)
144 prospective studies/ (607720)
145 prospective study/ (607720)
146 follow-up studies/ (528752)
147 follow up/ (0)
148 followup studies/ (0)
149 retrospective studies/ (1101096)
150 retrospective study/ (1101096)
151 case-control studies/ (321146)
152 exp case control study/ (1392397)
153 cross-sectional study/ (476776)
154 observational study/ (153290)
155 quasi experimental methods/ (0)
156 quasi experimental study/ (1072)
157 single-case studies as topic/ (100)
158 (observational study or validation studies or clinical study).pt. (159112)
159 (observational adj3 (study or studies or design or analysis or analyses)).ti,ab,kf. (193259)
160 cohort*.ti,ab,kf. (767738)
161 (prospective adj7 (study or studies or design or analysis or analyses)).ti,ab,kf. (443345)
162 ((follow up or followup) adj7 (study or studies or design or analysis or analyses)).ti,ab,kf. (133585)

163 ((longitudinal or longterm or (long adj term)) adj7 (study or studies or design or analysis or analyses or data)).ti,ab,kf. (286759)
164 (retrospective adj7 (study or studies or design or analysis or analyses or data or review)).ti,ab,kf. (573560)
165 ((case adj control) or (case adj comparison) or (case adj controlled)).ti,ab,kf. (134007)
166 (case-referent adj3 (study or studies or design or analysis or analyses)).ti,ab,kf. (356)
167 (population adj3 (study or studies or analysis or analyses)).ti,ab,kf. (197940)
168 (descriptive adj3 (study or studies or design or analysis or analyses)).ti,ab,kf. (91069)
169 ((multidimensional or (multi adj dimensional)) adj3 (study or studies or design or analysis or analyses)).ti,ab,kf. (3662)
170 (cross adj sectional adj7 (study or studies or design or research or analysis or analyses or survey or findings)).ti,ab,kf. (368567)
171 ((natural adj experiment) or (natural adj experiments)).ti,ab,kf. (2835)
172 (quasi adj (experiment or experiments or experimental)).ti,ab,kf. (16983)
173 ((non experiment or nonexperiment or non experimental or nonexperimental) adj3 (study or studies or design or analysis or analyses)).ti,ab,kf. (1474)
174 (prevalence adj3 (study or studies or analysis or analyses)).ti,ab,kf. (40588)
175 case series.ti,ab,kf. (84656)
176 case reports.pt. (1357461)
177 case reports as topic/ (58)
178 case report/ (0)
179 case study/ (1357461)
180 (case adj3 (report or reports or study or studies or histories)).ti,ab,kf. (622831)
181 organizational case studies.sh. (12631)
182 or/129-181 [Observational CADTH ML-EM-PsyInf https://searchfilters.cadth.ca/link/36] (5031684)
183 51 and 85 and 109 [SR results] (97)
184 (51 and 85 and 128) not 183 [RCT Results] (196)
185 (51 and 85 and 182) not (or/183-184) [Obs Results] (1503)
186 or/183-185 (1796)
187 limit 186 to english language (1735)

Database: Embase <1996 to 2024 March 18>
Search Date: 19 March 2024

----

1 *c reactive protein/ (26613)
2 (c-reactive protein or creactive protein).tw,kf,kw. (134608)
3 (crp adj3 (kit? or level? or measur* or test* or value?)).tw,kf,kw. (46697)
4 (crp adj3 (immunoassay or immunodiffusion or nephelometry or spectrophotometry)).tw,kf,kw. (225)
5 serum crp.tw,kf,kw. (5980)
6 or/1-5 [CRP] (151724)

7 *thorax radiography/ (12057)
8 (thora* adj4 (radiogra* or xray* or x-ray*)).tw,kf,kw. (6238)
9 thora* film*.tw,kf,kw. (10)
10 (chest adj4 (radiogra* or xray* or x-ray*)).tw,kf,kw. (78165)
11 chest film*.tw,kf,kw. (840)
12 CXR.tw,kf,kw. (9540)
13 or/7-12 [chest x-ray] (91220)
14 exp *echocardiography/ (67453)
15 (echocardiog* or echo-cardiog*).tw,kf,kw. (299128)
16 (cardiac adj2 (scan* or ultrasound*)).tw,kf,kw. (5668)
17 or/14-16 [echocardiogram] (309884)
18 *vital capacity/ (430)
19 vital capacit*.tw,kf,kw. (24989)
20 *forced expiratory volume/ (2787)
21 ((forced or timed) adj2 (expirat* volume* or vital capacit*)).tw,kf,kw. (36469)
22 *spirometry/ (5059)
23 (spiromet* or bronchospiromet*).tw,kf,kw. (44314)
24 ((lung? or pulmonar*) adj2 (function* or capacit*) adj2 (measur* or test*)).tw,kf,kw. (39409)
25 (diffus* capacit* adj3 carbon monoxide).tw,kf,kw. (3925)
26 (DLCO or TLCO).tw,kf,kw. (11805)
27 or/18-26 [lung function tests] (105646)
28 tomograph*.hw. and (exp *thorax/ or exp *lung/) (15387)
29 tomograph*.hw. and (cardiac or chest or lung* or pulmonary or thora*).tw,kf,kw. (332547)
30 tomograph*.tw,kf,kw. (648873)
31 ((ct or cat) adj5 (cardiac or chest or lung* or pulmonary or thora*)).tw,kf,kw. (104734)
32 or/28-31 [chest tomography] (863762)
33 natriuretic.hw. and (BNP or ntpprobnp or NT-proBNP or proBNP).tw,kf,kw. (33455)
34 ((BNP or ntpprobnp or NT-proBNP or proBNP) adj3 (level? or measur* or test* or value?)).tw,kf,kw. (22692)
35 or/33-34 [BNP test] (38848)
36 troponin.hw. and (level? or measur* or test* or value?).tw,kf,kw. (50399)
37 high sensitiv* troponin*.tw,kf,kw. (5839)
38 or/36-37 [troponin test] (51503)
39 *iron/ and exp *blood examination/ (77)
40 *iron/ and (level? or measur* or saturation or studies or study or test* or value?).tw,kf,kw. (35019)
41 ferritin*.hw. and (level? or measur* or saturation or studies or study or test* or value?).tw,kf,kw. (51884)
42 ((ferritin* or iron?) adj5 (level? or measur* or saturation or studies or study or test* or value?)).tw,kf,kw. (55736)
43 blood test*.tw,kf,kw. (48286)

44 or/39-43 [iron tests] (147234)
45 stand test*.tw,kf,kw. (4225)
46 standing test*.tw,kf,kw. (765)
47 or/45-46 [standing test] (4948)
48 (postexertion* malais* or post-exertion* malais*).tw,kf,kw. (410)
49 DPEMQ.mp. (0)
50 or/48-49 [DePaul test] (410)
51 6 or 13 or 17 or 27 or 32 or 35 or 38 or 44 or 47 or 50 [all tests] (1586749)
52 Long COVID/ [EMTREE] (6765)
53 ((chronic* or late sequela* or prolong*) adj2 (COVID or COVID-19 or COVID19 or coronavirus* or corona virus* or 2019-nCoV or 19nCoV or 2019nCoV or nCoV or n-CoV or "CoV 2" or CoV2 or SARS-CoV-2 or SARS-CoV2 or SARSCoV-2 or SARSCoV2 or SARS2 or SARS-2 or severe acute respiratory syndrome coronavirus 2 or 2019-novel CoV or Sars-coronavirus2 or Sars-coronavirus-2 or SARS-like coronavirus* or novel coronavirus* or novel corona virus* or novel CoV or OC43 or NL63 or 229E or HKU1 or HCoV* or Sars-coronavirus*)).ti,kw,kf. (2297)
54 ((long or longterm* or long-term* or "long haul" or "long haulers" or "long hauler" or long-haul*) adj (COVID or COVID-19 or COVID19 or coronavirus* or corona virus* or 2019-nCoV or 19nCoV or 2019nCoV or nCoV or n-CoV or "CoV 2" or CoV2 or SARS-CoV-2 or SARS-CoV2 or SARSCoV-2 or SARSCoV2 or SARS2 or SARS-2 or severe acute respiratory syndrome coronavirus 2 or 2019-novel CoV or Sars-coronavirus2 or Sars-coronavirus-2 or SARS-like coronavirus* or novel coronavirus* or novel corona virus* or novel CoV or OC43 or NL63 or 229E or HKU1 or HCoV* or Sars-coronavirus*)).ti,kw,kf. (5015)
55 ((after* or following or post* or "post acute") adj (COVID or COVID-19 or COVID19 or coronavirus* or corona virus* or 2019-nCoV or 19nCoV or 2019nCoV or nCoV or n-CoV or "CoV 2" or CoV2 or SARS-CoV-2 or SARS-CoV2 or SARSCoV-2 or SARSCoV2 or SARS2 or SARS-2 or severe acute respiratory syndrome coronavirus 2 or 2019-novel CoV or Sars-coronavirus2 or Sars-coronavirus-2 or SARS-like coronavirus* or novel coronavirus* or novel corona virus* or novel CoV or OC43 or NL63 or 229E or HKU1 or HCoV* or Sars-coronavirus*) adj3 (chronic* or clinical feature? or comorbid* or "co-morbid*" or complication* or condition* or consequence* or convalescen* or discharge* or disease* or disorder* or effect? or fatigue or followup* or follow-up* or function* or illness* or impact* or infection* or issue* or multimorbid* or "multi morbid*" or nonrecover* or non-recover* or persist* or prognos* or prolonged or recover* or recuperat* or recurr* or rehabilitat* or relaps* or residual or risk* or secondary or sequela* or sickness* or sign or signs or survivor* or survival* or syndrome* or symptom*)).ti,kw,kf. (5780)
56 or/52-55 [Part 1] (14416)
57 exp *severe acute respiratory syndrome coronavirus 2/ [EMTREE] (43096)
58 exp *coronavirus disease 2019/ [EMTREE] (302338)
59 SARS-CoV-2 variants.ti,hw,kf,kw. (2024)
60 (COVID-19 or COVID19).ti,kw,kf. (329775)
61 (((coronavirus* or corona virus*) and (hubei or wuhan or beijing or shanghai)) or (wuhan adj5 virus*)).ti,kw,kf. (670)
62 (2019-nCoV or 19nCoV or 2019nCoV or (nCoV or n-CoV or "CoV 2" or CoV2)).ti,kw,kf. (104928)
63 (SARS-CoV-2 or SARS-CoV2 or SARSCoV-2 or SARSCoV2 or SARS2 or SARS-2 or severe acute respiratory syndrome coronavirus 2).ti,kw,kf. (106852)
64 (2019-novel CoV or Sars-coronavirus2 or Sars-coronavirus-2 or SARS-like coronavirus* or ((novel or new or nouveau) adj2 (CoV or nCoV or covid or coronavirus* or corona virus or Pandemi*2)) or (coronavirus* and pneumonia)).ti,kw,kf. (8210)

65 (novel coronavirus* or novel corona virus* or novel CoV or ((coronavirus* or corona virus*) adj2 "2019") or ((coronavirus* or corona virus*) adj2 "19")).ti,kw,kf. (23632)
66 (coronavirus 2 or corona virus 2).ti,kw,kf. (6834)
67 (OC43 or NL63 or 229E or HKU1 or HCoV* or Sars-coronavirus*).ti,kw,kf. (2088)
68 (coronavirus* or corona virus* or COVID).ti. (320957)
69 or/57-68 [Covid 19] (410437)
70 ((long* or persist* or prolonged or residual) adj8 ((olfactory or chemosensor* or chemo-sensor*) adj (disorder* or dysfunction*))).ti,kw,kf. (109)
71 (((post acute or postacute or sub-acute or subacute or chronic) adj sequela*) or PASC or PACS).ti,kw,kf. (2795)
72 (post* adj3 (care or aftercare* or rehabilitati*) adj2 (clinic or clinics or centre or center or centres or centers or program*)).ti,kw,kf. (298)
73 ((post-intensive care or postintensive care or post-ICU) adj syndrome*).ti,kw,kf. (699)
74 (recovery adj2 (clinic or clinics or centre or center or centres or centers or program*)).ti,kw,kf. (1414)
75 ((chronic* or continuous* or continual* or continuing* or delay* or endur* or extend* or fluctuat* or gradual* or lasting* or legacy* or lengthy* or linger* or long* or "medium* term*" or mediumterm* or multisystem* or "multi system*" or ongoing* or permanent* or persist* or prolong* or protract* or relaps* or remission* or remit* or residual* or slow* or subacute* or "sub acute*") adj3 recover*).ti,kw,kf. (4935)
76 ((after discharg* or following discharg* or postacute* or "post acute*" or "post-acute" or postdischarg* or "post discharge" or "post discharging" or posthospital* or post-hospital* or postinfect* or "post infection" or "post infective*" or postviral* or "post viral*" or postvirus* or "post virus*" or postcritical or post-critical or postintensive or post-intensive or post-ICU) adj3 recover*).ti,kw,kf. (164)
77 ((chronic* or continuous* or continual* or continuing* or delay* or endur* or extend* or fluctuat* or gradual* or lasting* or legacy* or lengthy* or linger* or long* or "medium* term*" or mediumterm* or multisystem* or "multi system*" or ongoing or permanent* or persist* or prolong* or protract* or relaps* or remission* or remit* or residual* or slow* or subacute* or "sub acute*") adj3 (aftercare* or after-care* or complication? or condition* or consequence? or convalescen* or disabilit* or disease* or disorder* or feature* or illness* or issue* or prognos* or sequela* or sign or signs or suffering? or symptom* or syndrome* or recuperat*)).ti,kw,kf. (269600)
78 ((after discharg* or following discharg* or late-onset or postacute* or "post acute*" or postdischarg* or "post discharge" or "post discharging" or posthospital* or post-hospital* or postinfect* or "post infection" or "post infective*" or postviral* or "post viral*" or postvirus* or "post virus*" or postcritical or post-critical or postintensive or post-intensive or post-ICU or survivor* or survived) adj3 (complication? or condition* or consequence? or convalescen* or disabilit* or disease* or disorder* or feature* or illness* or issue* or prognos* or sequela* or sign or signs or suffering? or symptom* or syndrome* or recuperat*)).ti,kw,kf. (9403)
79 (nonrecover* or "non recover*" or "not recover*").ti,kw,kf. (201)
80 ("long* haul*" or long haul* or longhaul* or "long* tail*" or longtail* or longduration* or "long duration*" or longlast* or "long last*" or longstanding* or "long standing*" or "medium* term*" or mediumterm*).ti,kw,kf. (16293)
81 or/70-80 [Long term AE] (302070)
82 and/69,81 [Part 2] (9599)
83 56 or 82 [PCC/Long Covid Precise] (19917)
84 exp animals/ not exp humans/ (3624304)
85 (2019* or 202*).yr. (9382067)
86 (and/83,85) not 84 [PCC Long Covid Precise] (19835)
87 (systematic review or meta-analysis).pt. (0)

88 meta-analysis/ or systematic review/ or systematic reviews as topic/ or meta-analysis as topic/ or "meta analysis (topic)"/ or "systematic review (topic)"/ or exp technology assessment, biomedical/ or network meta-analysis/ (659044)
89 ((systematic* adj3 (review* or overview*)) or (methodologic* adj3 (review* or overview*))).ti,ab,kf. (427539)
90 ((quantitative adj3 (review* or overview* or synthes*)) or (research adj3 (integrati* or overview*))).ti,ab,kf. (18643)
91 ((integrative adj3 (review* or overview*)) or (collaborative adj3 (review* or overview*)) or (pool* adj3 analy*)).ti,ab,kf. (57591)
92 (data synthes* or data extraction* or data abstraction*).ti,ab,kf. (52190)
93 (handsearch* or hand search*).ti,ab,kf. (13959)
94 (mantel haenszel or peto or der simonian or dersimonian or fixed effect* or latin square*).ti,ab,kf. (47695)
95 (met analy* or metanaly* or technology assessment* or HTA or HTAs or technology overview* or technology appraisal*).ti,ab,kf. (20790)
96 (meta regression* or metaregression*).ti,ab,kf. (19452)
97 (meta-analy* or metaanaly* or systematic review* or biomedical technology assessment* or bio-medical technology assessment*).mp,hw. (780825)
98 (medline or cochrane or pubmed or medlars or embase or cinahl).ti,ab,hw. (480627)
99 (cochrane or (health adj2 technology assessment) or evidence report).jw. (31056)
100 (comparative adj3 (efficacy or effectiveness)).ti,ab,kf. (25665)
101 (outcomes research or relative effectiveness).ti,ab,kf. (15393)
102 ((indirect or indirect treatment or mixed-treatment or bayesian) adj3 comparison*).ti,ab,kf. (7753)
103 [(meta-analysis or systematic review).md.] (0)
104 (multi* adj3 treatment adj3 comparison*).ti,ab,kf. (420)
105 (mixed adj3 treatment adj3 (meta-analy* or metaanaly*)).ti,ab,kf. (260)
106 umbrella review*.ti,ab,kf. (1975)
107 (multi* adj2 paramet* adj2 evidence adj2 synthesis).ti,ab,kf. (34)
108 (multiparamet* adj2 evidence adj2 synthesis).ti,ab,kf. (22)
109 (multi-paramet* adj2 evidence adj2 synthesis).ti,ab,kf. (29)
110 or/87-109 [SR-MA-HTA- CADTH- ML-EM-PI https://searchfilters.cadth.ca/link/33] (1036615)
111 (Randomized Controlled Trial or Controlled Clinical Trial or Pragmatic Clinical Trial or Equivalence Trial or Clinical Trial, Phase III).pt. (0)
112 Randomized Controlled Trial/ (766262)
113 exp Randomized Controlled Trials as Topic/ (270313)
114 "Randomized Controlled Trial (topic)"/ (270187)
115 Controlled Clinical Trial/ (427110)
116 exp Controlled Clinical Trials as Topic/ (279663)
117 "Controlled Clinical Trial (topic)"/ (13406)
118 Randomization/ (90754)
119 Random Allocation/ (85546)
120 Double-Blind Method/ (165526)
121 Double Blind Procedure/ (190493)
122 Double-Blind Studies/ (174244)
123 Single-Blind Method/ (50435)

124 Single Blind Procedure/ (52501)
125 Single-Blind Studies/ (52501)
126 Placebos/ (300557)
127 Placebo/ (357442)
128 Control Groups/ (110480)
129 Control Group/ (110480)
130 (random* or sham or placebo*).ti,ab,hw,kf. (2418721)
131 ((singl* or doubl*) adj (blind* or dumm* or mask*)).ti,ab,hw,kf. (313482)
132 ((tripl* or trebl*) adj (blind* or dumm* or mask*)).ti,ab,hw,kf. (2238)
133 (control* adj3 (study or studies or trial* or group*)).ti,ab,kf. (1654602)
134 (Nonrandom* or non random* or non-random* or quasi-random* or quasirandom*).ti,ab,hw,kf. (67456)
135 allocated.ti,ab,hw. (104866)
136 ((open label or open-label) adj5 (study or studies or trial*)).ti,ab,hw,kf. (89854)
137 ((equivalence or superiority or non-inferiority or noninferiority) adj3 (study or studies or trial*)).ti,ab,hw,kf. (19264)
138 (pragmatic study or pragmatic studies).ti,ab,hw,kf. (976)
139 ((pragmatic or practical) adj3 trial*).ti,ab,hw,kf. (9156)
140 ((quasiexperimental or quasi-experimental) adj3 (study or studies or trial*)).ti,ab,hw,kf. (20864)
141 (phase adj3 (III or "3") adj3 (study or studies or trial*)).ti,hw,kf. (132858)
142 or/111-141 [CADTH RCT-ML-EM https://searchfilters.cadth.ca/link/122] (3545264)
143 epidemiologic methods.sh. (4)
144 epidemiologic studies.sh. (0)
145 observational study/ (363512)
146 observational studies as topic/ (363512)
147 clinical studies as topic/ (114971)
148 controlled before-after studies/ (143887)
149 cross-sectional studies/ (485434)
150 historically controlled study/ (154801)
151 interrupted time series analysis/ (136167)
152 exp seroepidemiologic studies/ (4554)
153 national longitudinal study of adolescent health/ (283)
154 cohort studies/ (986149)
155 cohort analysis/ (1124918)
156 longitudinal studies/ (180931)
157 longitudinal study/ (201366)
158 prospective studies/ (781374)
159 prospective study/ (888693)
160 follow-up studies/ (1623387)

161 follow up/ (2089912)
162 followup studies/ (0)
163 retrospective studies/ (1277476)
164 retrospective study/ (1559709)
165 case-control studies/ (175494)
166 exp case control study/ (228326)
167 cross-sectional study/ (614458)
168 observational study/ (363512)
169 quasi experimental methods/ (0)
170 quasi experimental study/ (12250)
171 single-case studies as topic/ (451)
172 (observational study or validation studies or clinical study).pt. (0)
173 (observational adj3 (study or studies or design or analysis or analyses)).ti,ab,kf. (369358)
174 cohort*.ti,ab,kf. (1531474)
175 (prospective adj7 (study or studies or design or analysis or analyses)).ti,ab,kf. (803384)
176 ((follow up or followup) adj7 (study or studies or design or analysis or analyses)).ti,ab,kf. (258642)
177 ((longitudinal or longterm or (long adj term)) adj7 (study or studies or design or analysis or analyses or data)).ti,ab,kf. (483844)
178 (retrospective adj7 (study or studies or design or analysis or analyses or data or review)).ti,ab,kf. (1181086)
179 ((case adj control) or (case adj comparison) or (case adj controlled)).ti,ab,kf. (209584)
180 (case-referent adj3 (study or studies or design or analysis or analyses)).ti,ab,kf. (430)
181 (population adj3 (study or studies or analysis or analyses)).ti,ab,kf. (354913)
182 (descriptive adj3 (study or studies or design or analysis or analyses)).ti,ab,kf. (174722)
183 ((multidimensional or (multi adj dimensional)) adj3 (study or studies or design or analysis or analyses)).ti,ab,kf. (5480)
184 (cross adj sectional adj7 (study or studies or design or research or analysis or analyses or survey or findings)).ti,ab,kf. (607180)
185 ((natural adj experiment) or (natural adj experiments)).ti,ab,kf. (3658)
186 (quasi adj (experiment or experiments or experimental)).ti,ab,kf. (26514)
187 ((non experiment or nonexperiment or non experimental or nonexperimental) adj3 (study or studies or design or analysis or analyses)).ti,ab,kf. (2378)
188 (prevalence adj3 (study or studies or analysis or analyses)).ti,ab,kf. (72043)
189 case series.ti,ab,kf. (154494)
190 case reports.pt. (0)
191 case reports as topic/ (159405)
192 case report/ (2200056)
193 case study/ (97236)
194 (case adj3 (report or reports or study or studies or histories)).ti,ab,kf. (1144771)
195 organizational case studies.sh. (2)
196 or/143-195 [Observational CADTH ML-EM-PsyInf https://searchfilters.cadth.ca/link/36] (8831787)

197 51 and 86 and 110 [SR results] (169)
198 51 and 86 and 142 [RCT results] (295)
199 51 and 86 and 196 [Obs results] (2749)
200 or/197-199 (2872)
201 limit 200 to embase (1858)
202 limit 201 to english language (1797)

Database: EBM Reviews - Cochrane Central Register of Controlled Trials <February 2024>
Search Date: 19 March 2024
----
1 c-reactive protein/ (6346)
2 (c-reactive protein or creactive protein).tw,kf,kw. (20803)
3 (crp adj3 (kit? or level? or measur* or test* or value?)).tw,kf,kw. (6340)
4 (crp adj3 (immunoassay or immunodiffusion or nephelometry or spectrophotometry)).tw,kf,kw. (43)
5 serum crp.tw,kf,kw. (709)
6 or/1-5 [CRP] (24441)
7 exp radiography, thoracic/ (490)
8 (thora* adj4 (radiogra* or xray* or x-ray*)).tw,kf,kw. (2010)
9 thora* film*.tw,kf,kw. (1)
10 (chest adj4 (radiogra* or xray* or x-ray*)).tw,kf,kw. (4575)
11 chest film*.tw,kf,kw. (31)
12 CXR.tw,kf,kw. (457)
13 or/7-12 [chest x-ray] (6064)
14 exp echocardiography/ (5260)
15 (echocardiog* or echo-cardiog*).tw,kf,kw. (16286)
16 (cardiac adj2 (scan* or ultrasound*)).tw,kf,kw. (501)
17 or/14-16 [echocardiogram] (17841)
18 vital capacity/ (2494)
19 vital capacit*.tw,kf,kw. (7509)
20 forced expiratory volume/ (6495)
21 ((forced or timed) adj2 (expirat* volume* or vital capacit*)).tw,kf,kw. (14075)
22 spirometry/ (2102)
23 (spiromet* or bronchospiromet*).tw,kf,kw. (8532)
24 ((lung? or pulmonar*) adj2 (function* or capacit*) adj2 (measur* or test*)).tw,kf,kw. (6299)
25 (diffus* capacit* adj3 carbon monoxide).tw,kf,kw. (483)
26 (DLCO or TLCO).tw,kf,kw. (1111)

27 or/18-26 [lung function tests] (28171)
28 tomograph*.hw. and (exp thorax/ or exp lung/) (550)
29 tomograph*.hw. and (cardiac or chest or lung* or pulmonary or thora*).tw,kf,kw. (6270)
30 tomograph*.tw,kf,kw. (32769)
31 ((ct or cat) adj5 (cardiac or chest or lung* or pulmonary or thora*)).tw,kf,kw. (4062)
32 or/28-31 [chest tomography] (35818)
33 natriuretic.hw. and (BNP or ntpprobnp or NT-proBNP or proBNP).tw,kf,kw. (1386)
34 ((BNP or ntpprobnp or NT-proBNP or proBNP) adj3 (level? or measur* or test* or value?)).tw,kf,kw. (2356)
35 or/33-34 [BNP test] (2941)
36 troponin.hw. and (level? or measur* or test* or value?).tw,kf,kw. (1493)
37 high sensitiv* troponin*.tw,kf,kw. (532)
38 or/36-37 [troponin test] (1895)
39 exp iron/ and hematological tests/ (23)
40 exp iron/ and (level? or measur* or saturation or studies or study or test* or value?).tw,kf,kw. (2845)
41 ferritins.hw. and (level? or measur* or saturation or studies or study or test* or value?).tw,kf,kw. (1280)
42 ((ferritin* or iron?) adj5 (level? or measur* or saturation or studies or study or test* or value?)).tw,kf,kw. (5938)
43 blood test*.tw,kf,kw. (8068)
44 or/39-43 [iron tests] (15232)
45 stand test*.tw,kf,kw. (2003)
46 standing test*.tw,kf,kw. (199)
47 or/45-46 [standing test] (2188)
48 (postexertion* malais* or post-exertion* malais*).tw,kf,kw. (58)
49 DPEMQ.mp. (0)
50 or/48-49 [DePaul test] (58)
51 6 or 13 or 17 or 27 or 32 or 35 or 38 or 44 or 47 or 50 [all tests] (124194)
52 Post-Acute COVID-19 Syndrome/ (186)
53 ((chronic* or late sequela* or prolong*) adj2 (COVID or COVID-19 or COVID19 or coronavirus* or corona virus* or 2019-nCoV or 19nCoV or 2019nCoV or nCoV or n-CoV or "CoV 2" or CoV2 or SARS-CoV-2 or SARS-CoV2 or SARSCoV-2 or SARSCoV2 or SARS2 or SARS-2 or severe acute respiratory syndrome coronavirus 2 or 2019-novel CoV or Sars-coronavirus2 or Sars-coronavirus-2 or SARS-like coronavirus* or novel coronavirus* or novel corona virus* or novel CoV or OC43 or NL63 or 229E or HKU1 or HCoV* or Sars-coronavirus*)).tw,kw,kf. (53)
54 ((long or longterm* or long-term* or "long haul" or "long haulers" or "long hauler" or long-haul*) adj (COVID or COVID-19 or COVID19 or coronavirus* or corona virus* or 2019-nCoV or 19nCoV or 2019nCoV or nCoV or n-CoV or "CoV 2" or CoV2 or SARS-CoV-2 or SARS-CoV2 or SARSCoV-2 or SARSCoV2 or SARS2 or SARS-2 or severe acute respiratory syndrome coronavirus 2 or 2019-novel CoV or Sars-coronavirus2 or Sars-coronavirus-2 or SARS-like coronavirus* or novel coronavirus* or novel corona virus* or novel CoV or OC43 or NL63 or 229E or HKU1 or HCoV* or Sars-coronavirus*)).tw,kw,kf. (389)
55 ((after* or following or post* or "post acute") adj (COVID or COVID-19 or COVID19 or coronavirus* or corona virus* or 2019-nCoV or 19nCoV or 2019nCoV or nCoV or n-CoV or "CoV 2" or CoV2 or SARS-CoV-2 or SARS-CoV2 or SARSCoV-2 or SARSCoV2 or SARS2 or SARS-2 or severe acute

respiratory syndrome coronavirus 2 or 2019-novel CoV or Sars-coronavirus2 or Sars-coronavirus-2 or SARS-like coronavirus* or novel coronavirus* or novel corona virus* or novel CoV or OC43 or NL63 or 229E or HKU1 or HCoV* or Sars-coronavirus*) adj3 (chronic* or clinical feature? or comorbid* or "co-morbid*" or complication* or condition* or consequence* or convalescen* or discharge* or disease* or disorder* or effect? or fatigue or followup* or follow-up* or function* or illness* or impact* or infection* or issue* or multimorbid* or "multi morbid*" or nonrecover* or non-recover* or persist* or prognos* or prolonged or recover* or recuperat* or recurr* or rehabilitat* or relaps* or residual or risk* or secondary or sequela* or sickness* or sign or signs or survivor* or survival* or syndrome* or symptom*)).tw,kw,kf. (636)
56 or/52-55 [PCC -Long Covid Set1] (925)
57 exp Covid-19/ (7402)
58 SARS-CoV-2/ (3077)
59 SARS-CoV-2 variants.mp. (129)
60 (COVID-19 or COVID19).tw,kw,kf. (18324)
61 (((coronavirus* or corona virus*) and (hubei or wuhan or beijing or shanghai)) or (wuhan adj5 virus*) or (2019-nCoV or 19nCoV or 2019nCoV) or (nCoV or n-CoV or "CoV 2" or CoV2)).tw,kw,kf. (6055)
62 (SARS-CoV-2 or SARS-CoV2 or SARSCoV-2 or SARSCoV2 or SARS2 or SARS-2 or severe acute respiratory syndrome coronavirus 2).tw,kw,kf. (6085)
63 (2019-novel CoV or Sars-coronavirus2 or Sars-coronavirus-2 or SARS-like coronavirus* or ((novel or new or nouveau) adj2 (CoV or nCoV or covid or coronavirus* or corona virus or Pandemi*2)) or (coronavirus* and pneumonia)).tw,kw,kf. (2404)
64 (novel coronavirus* or novel corona virus* or novel CoV).tw,kw,kf. (901)
65 (((coronavirus* or corona virus*) adj2 "2019") or ((coronavirus* or corona virus*) adj2 "19") or (coronavirus 2 or corona virus 2)).tw,kw,kf. (9197)
66 (OC43 or NL63 or 229E or HKU1 or HCoV* or Sars-coronavirus*).tw,kw,kf. (139)
67 [(COVID-19 or severe acute respiratory syndrome coronavirus 2).os.] (0)
68 (coronavirus* or corona virus* or COVID).ti. (13152)
69 or/57-68 [COVID-19] (20248)
70 ((long* or persist* or prolonged or residual) adj8 ((olfactory or chemosensor* or chemo-sensor*) adj (disorder* or dysfunction*))).tw,kw,kf. (33)
71 (((post acute or postacute or sub-acute or subacute or chronic) adj sequela*) or PASC or PACS).tw,kw,kf. (508)
72 (post* adj3 (care or aftercare* or rehabilitati*) adj2 (clinic or clinics or centre or center or centres or centers or program*)).tw,kw,kf. (489)
73 ((post-intensive care or postintensive care or post-ICU) adj syndrome*).tw,kw,kf. (105)
74 (recovery adj2 (clinic or clinics or centre or center or centres or centers or program*)).tw,kw,kf. (605)
75 ((chronic* or continuous* or continual* or continuing* or delay* or endur* or extend* or fluctuat* or gradual* or lasting* or legacy* or lengthy* or linger* or long* or "medium* term*" or mediumterm* or multisystem* or "multi system*" or ongoing* or permanent* or persist* or prolong* or protract* or relaps* or remission* or remit* or residual* or slow* or subacute* or "sub acute*") adj3 recover*).tw,kw,kf. (4940)
76 ((after discharg* or following discharg* or postacute* or "post acute*" or "post-acute" or postdischarg* or "post discharge" or "post discharging" or posthospital* or post-hospital* or postinfect* or "post infection" or "post infective*" or postviral* or "post viral*" or postvirus* or "post virus*" or postcritical or post-critical or postintensive or post-intensive or post-ICU) adj3 recover*).tw,kw,kf. (179)
77 ((chronic* or continuous* or continual* or continuing* or delay* or endur* or extend* or fluctuat* or gradual* or lasting* or legacy* or lengthy* or linger* or long* or "medium* term*" or mediumterm* or multisystem* or "multi system*" or ongoing or permanent* or persist* or prolong* or protract* or relaps* or remission* or remit* or residual* or slow* or subacute* or "sub acute*") adj3 (aftercare* or after-care* or complication? or condition* or

consequence? or convalescen* or disabilit* or disease* or disorder* or feature* or illness* or issue* or prognos* or sequela* or sign or signs or suffering? or symptom* or syndrome* or recuperat*)).tw,kw,kf. (115440)
78 ((after discharg* or following discharg* or late-onset or postacute* or "post acute*" or postdischarg* or "post discharge" or "post discharging" or posthospital* or post-hospital* or postinfect* or "post infection" or "post infective*" or postviral* or "post viral*" or postvirus* or "post virus*" or postcritical or post-critical or postintensive or post-intensive or post-ICU or survivor* or survived) adj3 (complication? or condition* or consequence? or convalescen* or disabilit* or disease* or disorder* or feature* or illness* or issue* or prognos* or sequela* or sign or signs or suffering? or symptom* or syndrome* or recuperat*)).tw,kw,kf. (2473)
79 (nonrecover* or "non recover*" or "not recover*" or ("long* haul*" or long haul* or longhaul* or "long* tail*" or longtail* or longduration* or "long duration*" or longlast* or "long last*" or longstanding* or "long standing*" or "medium* term*" or mediumterm*)).tw,kw,kf. (9811)
80 or/70-79 [Long haul terms] (131386)
81 69 and 80 [COVID-19 AND Long Haul: Part 2 of PCC Strategy] (2110)
82 Long Term Adverse Effects/ and 69 [Part 3 of PCC sensitive strategy] (0)
83 exp animals/ not exp humans/ (3681)
84 (2019* or 202*).yr. (575884)
85 ((or/56,81-82) and 84) not 83 [PCC/Long Covid Sensitive-ML] (2516)
86 51 and 85 (429)
87 limit 86 to english language (425)

Supplementary File S3. *PRISMA flow chart*

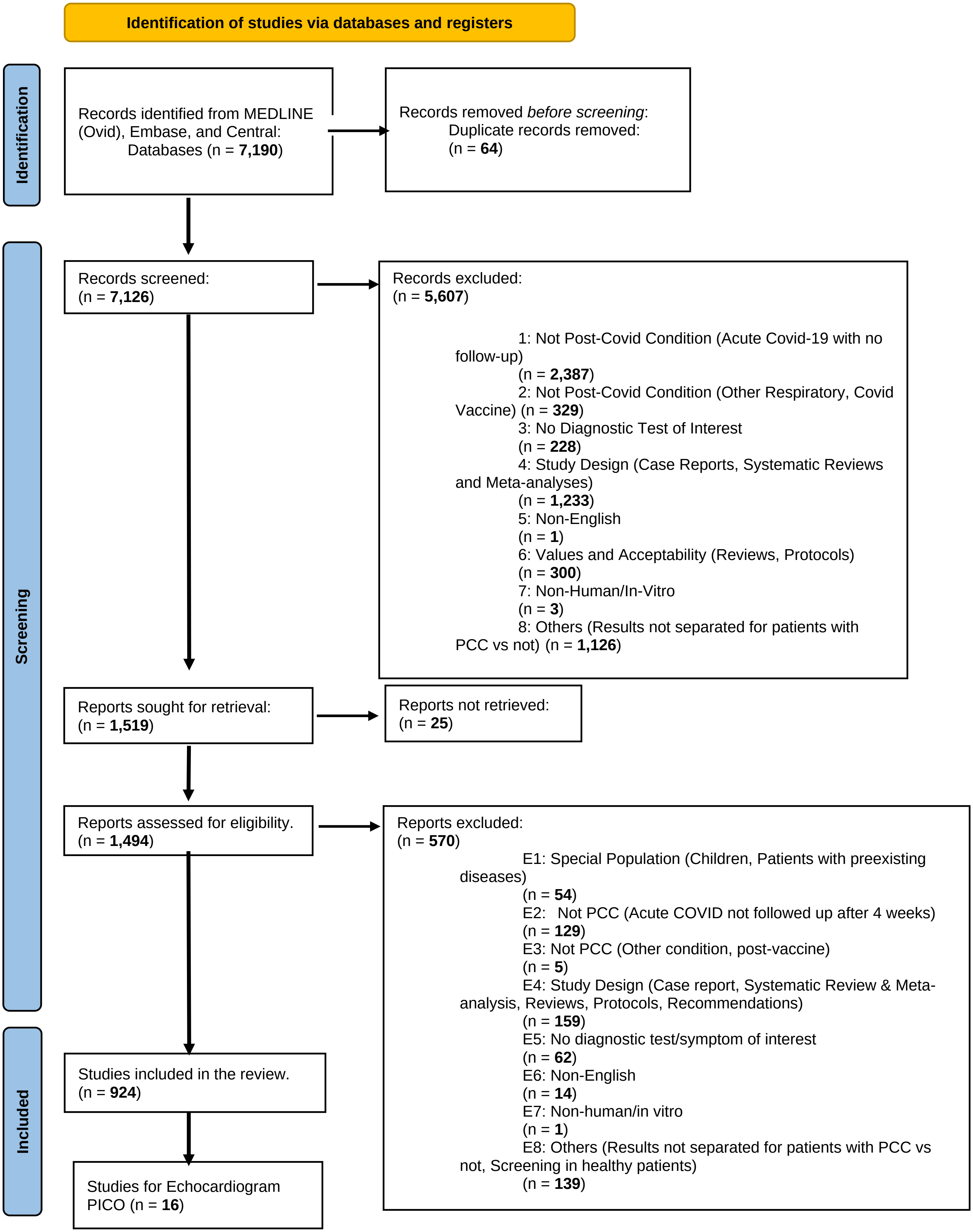

Supplementary File S4: *Forest Plots*

Supplementary Figure S1.a: Forest plot showing mean difference in LVEF between hospitalized patients with PCC and patients without PCC.

	Patients with PCC			Patients without PCC				Mean difference
Study or Subgroup	Mean	SD	Total	Mean	SD	Total	Weight	IV, Random, 95% CI
Acanfora 2022	61.9	13.7	30	70.4	5.7	20	1.5%	-8.50 [-14.00 , -3.00]
Ceccheto 2023	60.75	1.37	115	61	1.58	110	21.1%	-0.25 [-0.64 , 0.14]
Duran 2022	59.4	2.6	102	61.6	3.7	100	16.4%	-2.20 [-3.08 , -1.32]
Gryglewska-Wawrzak 2023	61.25	2.05	44	62.5	1.6	102	18.5%	-1.25 [-1.93 , -0.57]
Luchian 2022	55.7	4.6	23	57.6	4.5	43	6.4%	-1.90 [-4.21 , 0.41]
Niebauer 2023	60.51	6.81	113	61.21	6.34	37	6.0%	-0.70 [-3.10 , 1.70]
Rajotiya 2024	57.73	2.47	23	59.45	2.18	20	11.7%	-1.72 [-3.11 , -0.33]
Tas 2023	61.5	2.22	51	62	1.6	95	18.4%	-0.50 [-1.19 , 0.19]
**Total (Wald[a])**			**501**			**527**	**100.0%**	**-1.23 [-1.92 , -0.54]**

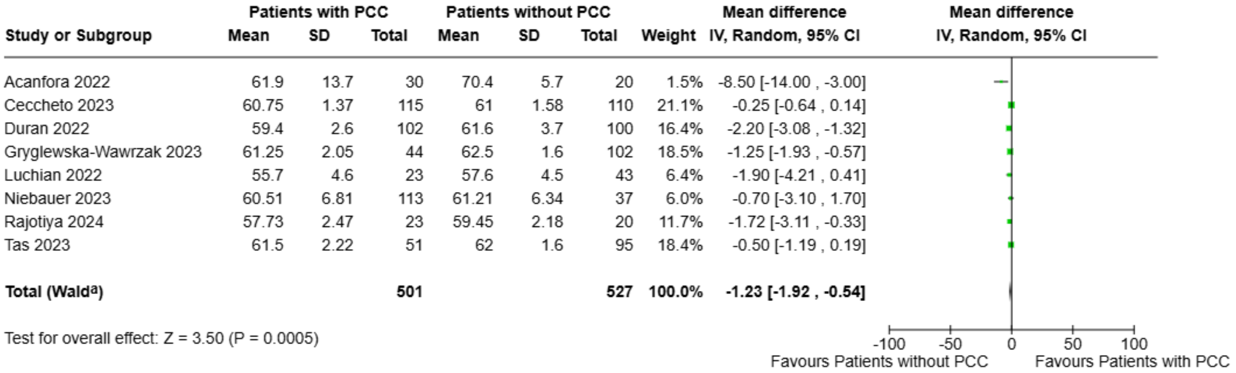

Test for overall effect: Z = 3.50 (P = 0.0005)

Heterogeneity: Tau^2 (REML[b]) = 0.55; Chi^2 = 29.78, df = 7 (P = 0.0001); I^2 = 73%

Footnotes

[a]CI calculated by Wald-type method.

[b]Tau^2 calculated by Restricted Maximum-Likelihood method.

Supplementary Figure S1.b: Forest plot showing mean difference in LVEF between non- hospitalized patients with PCC and patients without PCC.

	Patients with PCC			Patients without PCC				Mean difference
Study or Subgroup	Mean	SD	Total	Mean	SD	Total	Weight	IV, Fixed, 95% CI
Chudzik 2022	59.5	0.36	218	60	0.0001	270	100.0%	-0.50 [-0.55 , -0.45]
**Total**			**218**			**270**	**100.0%**	**-0.50 [-0.55 , -0.45]**

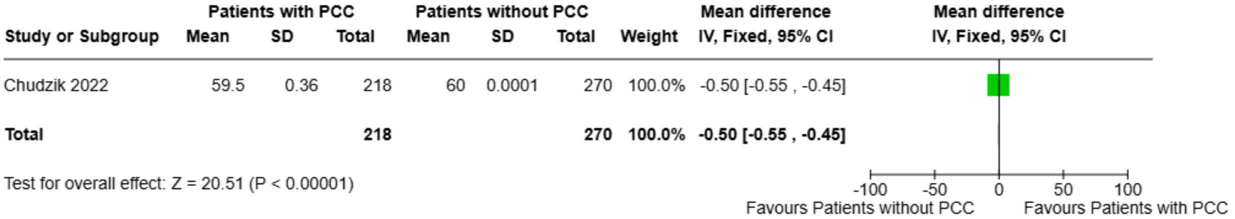

Test for overall effect: Z = 20.51 (P < 0.00001)

Heterogeneity: Not applicable

Supplementary Figure S1.c: Forest plot showing mean difference in LVEF between mixed-setting patients with PCC and patients without PCC.

	Patients with PCC			Patients without PCC				Mean difference
Study or Subgroup	Mean	SD	Total	Mean	SD	Total	Weight	IV, Fixed, 95% CI
Aparisi 2021	61.25	2.05	44	62.5	1.6	102	93.4%	-1.25 [-1.93 , -0.57]
Durstenfeld 2022	64	7.2	47	63.7	5.8	55	6.6%	0.30 [-2.27 , 2.87]
**Total**			**91**			**157**	**100.0%**	**-1.15 [-1.81 , -0.49]**

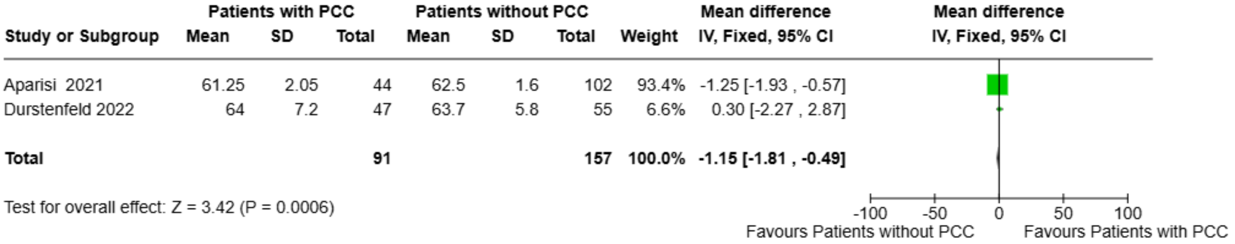

Test for overall effect: Z = 3.42 (P = 0.0006)

Heterogeneity: Chi^2 = 1.31, df = 1 (P = 0.25); I^2 = 24%

Supplementary Figure S1.d: Forest plot showing mean difference in LVEDV between hospitalized patients with PCC and patients without PCC.

	Patients with PCC			Patients without PCC				Mean difference
Study or Subgroup	Mean	SD	Total	Mean	SD	Total	Weight	IV, Fixed, 95% CI
Acanfora 2022	114.6	52.5	30	94.1	27.9	20	1.2%	20.50 [-1.92 , 42.92]
Gryglewska-Wawrzak 2023	88	7.29	44	89.75	5.98	102	98.8%	-1.75 [-4.20 , 0.70]
**Total**			**74**			**122**	**100.0%**	**-1.49 [-3.92 , 0.94]**

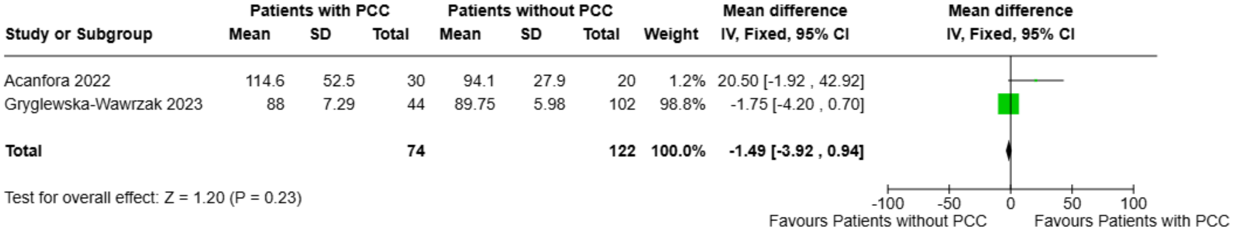

Test for overall effect: Z = 1.20 (P = 0.23)

Heterogeneity: Chi^2 = 3.74, df = 1 (P = 0.05); I^2 = 73%

Supplementary Figure S1.e: Forest plot showing LVEF less than 50% between hospitalized patients with PCC and patients without PCC.

	Patients with PCC		Patients without PCC			Odds ratio
Study or Subgroup	Events	Total	Events	Total	Weight	IV, Fixed, 95% CI
Jutant 2022	6	40	4	43	100.0%	1.72 [0.45 , 6.61]
**Total**		**40**		**43**	**100.0%**	**1.72 [0.45 , 6.61]**
Total events:	6		4			

Test for overall effect: Z = 0.79 (P = 0.43)

Heterogeneity: Not applicable

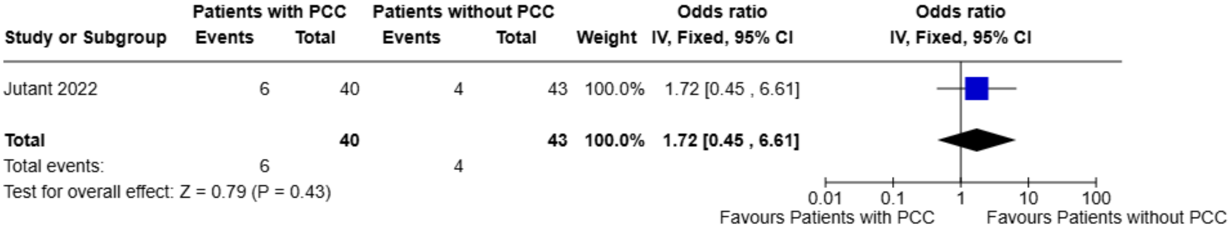

Supplementary Figure S2.a: Forest plot showing mean difference in TAPSE between hospitalized patients with PCC and patients without PCC.

	Patients with PCC			Patients without PCC				Mean difference
Study or Subgroup	Mean	SD	Total	Mean	SD	Total	Weight	IV, Random, 95% CI
Ceccheto 2023	21.67	3.75	115	22	3	110	17.6%	-0.33 [-1.22 , 0.56]
Ceccheto 2023 (2)	22.3	3.7	144	21.67	3	81	17.6%	0.63 [-0.26 , 1.52]
Duran 2022	20.8	0.2	102	23.3	0.26	100	20.2%	-2.50 [-2.56 , -2.44]
Gryglewska-Wawrzak 2023	23.31	3.83	44	22.83	3.98	102	14.9%	0.48 [-0.89 , 1.85]
Luchian 2022	24.5	4.9	23	25	4	43	10.0%	-0.50 [-2.83 , 1.83]
Tas 2023	2.3	0.83	51	2.46	1.129	95	19.8%	-0.16 [-0.48 , 0.16]
**Total (Wald[a])**			**479**			**531**	**100.0%**	**-0.46 [-1.50 , 0.57]**

Test for overall effect: Z = 0.88 (P = 0.38)

Heterogeneity: Tau² (REML[b]) = 1.38; Chi² = 280.29, df = 5 (P < 0.00001); I² = 96%

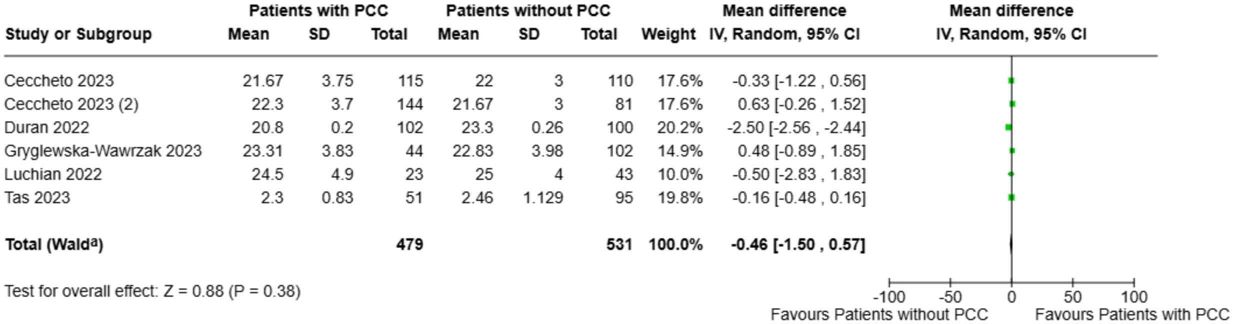

Footnotes

[a]CI calculated by Wald-type method.

[b]Tau² calculated by Restricted Maximum-Likelihood method.

Supplementary Figure S2.b: Forest plot showing mean difference in TAPSE between mixed-setting patients with PCC and patients without PCC.

	Patients with PCC			Patients without PCC				Mean difference
Study or Subgroup	Mean	SD	Total	Mean	SD	Total	Weight	IV, Random, 95% CI
Aparisi 2021	23.33	5.37	41	23.33	2.34	29	17.6%	0.00 [-1.85 , 1.85]
Aparisi 202	22.75	1.62	10	22.75	1.08	60	55.8%	0.00 [-1.04 , 1.04]
Durstenfeld 2022	22.5	4.1	47	22.9	3.6	55	26.5%	-0.40 [-1.91 , 1.11]
**Total (Wald[a])**			**98**			**144**	**100.0%**	**-0.11 [-0.88 , 0.67]**

Test for overall effect: Z = 0.27 (P = 0.79)

Heterogeneity: Tau² (REML[b]) = 0.00; Chi² = 0.20, df = 2 (P = 0.91); I² = 0%

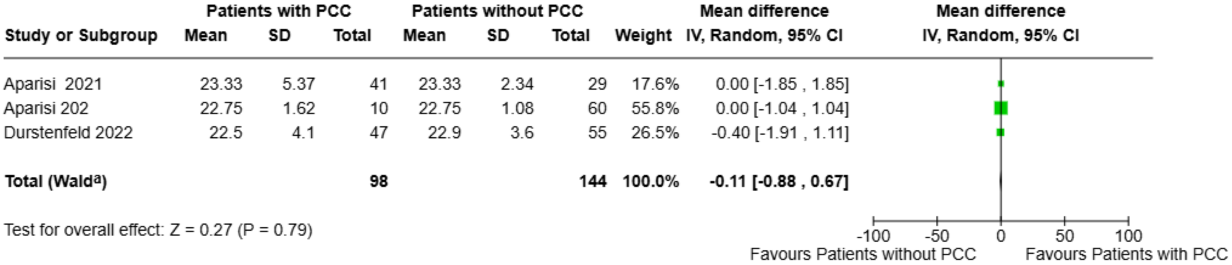

Footnotes

[a]CI calculated by Wald-type method.

[b]Tau² calculated by Restricted Maximum-Likelihood method.

Supplementary Figure S3.a: Forest plot showing mean difference in right atrial diameter between hospitalized patients with PCC and patients without PCC.

	Patients with PCC			Patients without PCC				Mean difference
Study or Subgroup	Mean	SD	Total	Mean	SD	Total	Weight	IV, Fixed, 95% CI
Duran 2022	32.9	4.3	102	30.8	4.7	100	100.0%	2.10 [0.86 , 3.34]
**Total**			**102**			**100**	**100.0%**	**2.10 [0.86 , 3.34]**

Test for overall effect: Z = 3.31 (P = 0.0009)

Heterogeneity: Not applicable

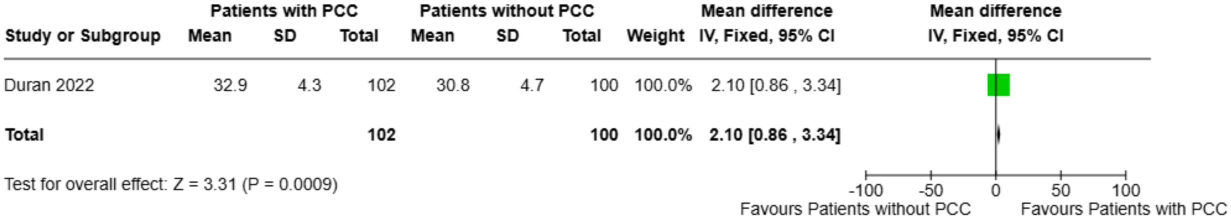

Supplementary Figure S3.b: Forest plot showing mean difference in RVSP between mixed-setting patients with PCC and patients without PCC.

Study or Subgroup	Patients with PCC Mean	SD	Total	Patients without PCC Mean	SD	Total	Weight	Mean difference IV, Fixed, 95% CI
Aparisi 2021	22	1.85	41	16.75	1.73	29	100.0%	5.25 [4.40 , 6.10]
**Total**			**41**			**29**	**100.0%**	**5.25 [4.40 , 6.10]**

Test for overall effect: Z = 12.15 (P < 0.00001)

Heterogeneity: Not applicable

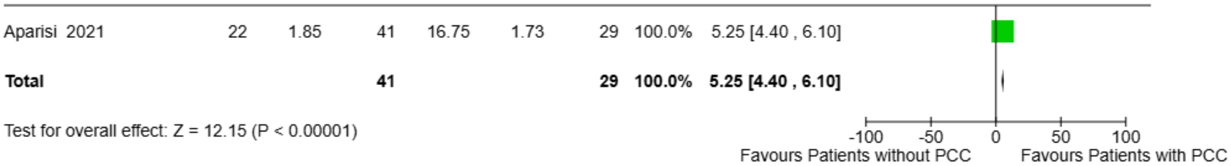

Supplementary Figure S3.c: Forest plot showing mean difference in right ventricular diameter between hospitalized patients with PCC and patients without PCC.

Study or Subgroup	Patients with PCC Mean	SD	Total	Patients without PCC Mean	SD	Total	Weight	Mean difference IV, Fixed, 95% CI
Tas 2023	34.5	4.9	51	33.8	5	95	100.0%	0.70 [-0.98 , 2.38]
**Total**			**51**			**95**	**100.0%**	**0.70 [-0.98 , 2.38]**

Test for overall effect: Z = 0.82 (P = 0.41)

Heterogeneity: Not applicable

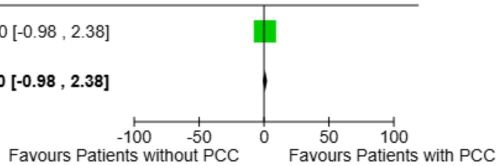

Supplementary Figure S3.d: Forest plot showing mean difference in right ventricular diameter between non-hospitalized patients with PCC and patients without PCC.

Study or Subgroup	Patients with PCC Mean	SD	Total	Patients without PCC Mean	SD	Total	Weight	Mean difference IV, Fixed, 95% CI
Chudzik 2022	28	2.98	218	28	2.98	270	100.0%	0.00 [-0.53 , 0.53]
**Total**			**218**			**270**	**100.0%**	**0.00 [-0.53 , 0.53]**

Test for overall effect: Z = 0.00 (P = 1.00)

Heterogeneity: Not applicable

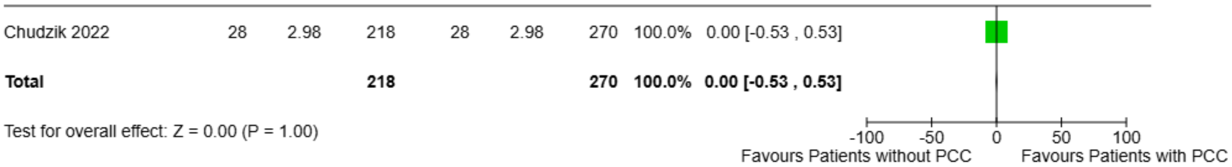

Supplementary Figure S4: Forest plot showing pericardial effusion between mixed-setting patients with PCC and patients without PCC.

Study or Subgroup	Patients with PCC Events	Total	Patients without PCC Events	Total	Weight	Risk difference IV, Fixed, 95% CI
Durstenfeld 2022	4	47	0	55	100.0%	0.09 [-0.00 , 0.17]
**Total**		**47**		**55**	**100.0%**	**0.09 [-0.00 , 0.17]**
Total events:	4		0			

Test for overall effect: Z = 1.94 (P = 0.05)

Heterogeneity: Not applicable

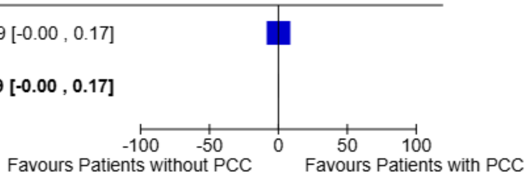

Supplement: Supplementary file 1 [file jcm-15-04643-s001.zip › jcm-4303893-supplementary.pdf]
